# A novel 6-metabolite signature for prediction of clinical outcomes in type 2 diabetic patients undergoing percutaneous coronary intervention

**DOI:** 10.1186/s12933-022-01561-1

**Published:** 2022-07-04

**Authors:** Xue-bin Wang, Ning-hua Cui, Xia’nan Liu

**Affiliations:** 1 Department of Clinical Laboratory, Key Clinical Laboratory of Henan Province, The First Affiliated Hospital of Zhengzhou University, Jianshe East Road No.1, Zhengzhou, 450000 Henan China; 2 Zhengzhou Key Laboratory of Children’s Infection and Immunity, Children’s Hospital Affiliated to Zhengzhou University, Zhengzhou, 450000 Henan China

**Keywords:** Metabolomics, Type 2 diabetes, Clinical outcomes after PCI, Prediction model, NAD metabolites

## Abstract

**Background:**

Outcome prediction tools for patients with type 2 diabetes mellitus (T2DM) undergoing percutaneous coronary intervention (PCI) are lacking. Here, we developed a machine learning-based metabolite classifier for predicting 1-year major adverse cardiovascular events (MACEs) after PCI among patients with T2DM.

**Methods:**

Serum metabolomic profiling was performed in a nested case–control study of 108 matched pairs of patients with T2DM occurring and not occurring MACEs at 1 year after PCI, then the matched pairs were 1:1 assigned into the discovery and internal validation sets. External validation was conducted using targeted metabolite analyses in an independent prospective cohort of 301 patients with T2DM receiving PCI. The function of candidate metabolites was explored in high glucose-cultured human aortic smooth muscle cells (HASMCs).

**Results:**

Overall, serum metabolome profiles differed between diabetic patients with and without 1-year MACEs after PCI. Through VSURF, a machine learning approach for feature selection, we identified the 6 most important metabolic predictors, which mainly targeted the nicotinamide adenine dinucleotide (NAD^+^) metabolism. The 6-metabolite model based on random forest and XGBoost algorithms yielded an area under the curve (AUC) of ≥ 0.90 for predicting MACEs in both discovery and internal validation sets. External validation of the 6-metabolite classifier also showed good accuracy in predicting MACEs (AUC 0.94, 95% CI 0.91–0.97) and target lesion failure (AUC 0.89, 95% CI 0.83–0.95). In vitro, there were significant impacts of altering NAD^+^ biosynthesis on bioenergetic profiles, inflammation and proliferation of HASMCs.

**Conclusion:**

The 6-metabolite model may help for noninvasive prediction of 1-year MACEs following PCI among patients with T2DM.

**Supplementary Information:**

The online version contains supplementary material available at 10.1186/s12933-022-01561-1.

## Background

Patients with type 2 diabetes mellitus (T2DM) account for more than a quarter of all coronary artery disease (CAD) patients receiving percutaneous coronary intervention (PCI) [1]. Despite great advances in stent technologies, T2DM remains a strong indicator of major adverse cardiovascular events (MACEs) after PCI [2, 3]. Thus, identifying biomarkers for noninvasive prediction of post-PCI outcomes among type 2 diabetic patients has substantial clinical implications [4].

Metabolomics, which provides untargeted measurements of the multiparametric metabolic response of living systems to pathophysiological stimuli [5], has the potential to both discover new biomarkers and reveal key metabolic pathways intrinsic to the disease pathogenesis [6]. Although such metabolomic approaches have been increasingly explored in cardiovascular biomarker discovery, most previous studies have concentrated on the screening of metabolic biomarkers for the discrimination of established CAD from non-CAD controls [7, 8]. Yet it remains unclear how systemic metabolic alterations impact clinical outcomes after PCI, especially for patients having T2DM. Moreover, the majority of metabolic biomarker candidates for cardiovascular disease were identified using the classical generalized linear method of regression [9, 10]. Modern machine leaning approaches, which are better able to incorporate high-order nonlinear associations between predictors to gain predictive performance [11], have rarely been applied to outcome predictions for type 2 diabetic patients receiving PCI.

Hence, in a nested case–control study of 216 patients with T2DM who underwent PCI due to obstructive CAD, we first assessed the prospective associations of serum metabolic profiles at baseline with the risk of incident MACEs at 1 year after PCI. Then, a 6-metabolite model, mainly targeting the pathway of nicotinamide adenine dinucleotide (NAD^+^) metabolism, was developed and internally validated for the prediction of 1-year MACEs following PCI based on a set of machine learning algorithms. Next, we externally verified the 6-metabolite model using a targeted metabolite analysis in an independent prospective cohort of 301 diabetic patients who received PCI. Finally, we explored the biological relevance of altering NAD^+^ biosynthesis to abnormal phenotypes of human aortic smooth muscle cells (HASMCs) under high glucose (HG) conditions.

## Methods

### Study design and participants

**Fig. 1:**
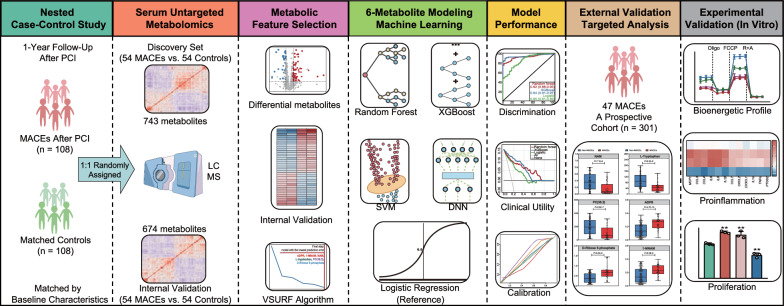
Study workflow and design. *PCI* percutaneous coronary intervention; *MACEs* major adverse cardiovascular events; *VSURF* variable selection using random forest; *XGBoost* extreme gradient boosting; *SVM* Support Vector Machines; *DNN* deep neural network

An overview of the study design is depicted in Fig. 1. In brief, we first conducted a nested case–control study within a prospective cohort of 702 patients with T2DM who underwent primary PCI from Sep 2017 to Jan 2019 in the First Affiliated Hospital of Zhengzhou University. As previously described [12, 13], the prospective cohort excluded patients who had systematic diseases including cancer, serious infection, chronic liver disease, and type 1 diabetes. T2DM was diagnosed based on the 2014 American Diabetes Association criteria [14]. All participants were hospitalized for angiographically confirmed obstructive CAD [15] and underwent PCI at baseline, and then completed a follow-up of 1 year to track MACEs [composite of all-cause death, myocardial infarction (MI), stroke, and repeat revascularization] as the primary outcome. Clinical, angiographic, and procedural data were collected at baseline (Table 1), and outcome data were obtained from medical records and telephone interviews with participants at 30 days and 6, 9, 12 months after PCI. Within a median follow up of 12.5 months (interquartile range [IQR]: 11.9–12.6 months), 108 (15.4%) patients occurred MACEs (all-cause death, n = 46; repeat revascularization, n = 54; MI, n = 13; stroke, n = 7). Of the remaining 594 participants without the occurrence of MACEs, 108 individuals, matched for baseline characteristics using propensity scores [16], were selected as the controls. Then, the matched case–control pairs were randomly assigned (1:1) to a discovery set or an internal validation set. Both sets could provide a > 90% power to detect a fold change of > 4/3 or < 3/4 for differential metabolites at a false discovery rate (FDR) of < 5%.

**Table 1:** Baseline characteristics of each study set

Variables^a^	Nested case–control study						Prospective cohort study		
	Discovery set			Internal validation set			External validation set		
	MACEs (n = 54)	Controls (n = 54)	*P*^a^	MACEs (n = 54)	Controls (n = 54)	*P*^a^	With MACEs (n = 47)	Without MACEs (n = 254)	*P*^a^
Clinical data									
Age, years	63.4 ± 9.5	63.1 ± 9.5	0.86	63.9 ± 9.0	64.5 ± 10.6	0.78	62.3 ± 9.5	62.8 ± 9.4	0.71
Male	33 (61.1)	35 (64.8)	0.69	32 (59.3)	34 (63.0)	0.69	30 (63.8)	146 (57.5)	0.42
Current smokers	11 (20.4)	11 (20.4)	1.00	10 (18.5)	12 (22.2)	0.63	17 (36.2)	53 (20.9)	**0.023**
BMI, kg/m^2,^	25.6 ± 4.0	25.3 ± 4.3	0.73	25.1 ± 4.5	25.4 ± 3.6	0.75	25.2 ± 3.9	25.0 ± 4.1	0.75
FPG, mmol/L	8.8 ± 2.1	8.4 ± 1.9	0.24	8.4 ± 2.1	8.8 ± 2.1	0.37	8.2 ± 2.3	8.4 ± 2.1	0.72
HbA1c, (%)	7.6 ± 1.8	7.3 ± 1.5	0.33	7.2 ± 1.7	7.8 ± 2.3	0.15	8.1 ± 1.9	7.4 ± 1.7	**0.020**
Diabetes duration, years	9.2 ± 5.1	10.0 ± 4.8	0.39	9.9 ± 5.4	9.0 ± 4.9	0.36	9.5 ± 5.6	10.0 ± 5.4	0.58
Diabetes management									
Lifestyle modification	12 (22.2)	18 (33.3)	0.55	15 (27.8)	17 (31.5)	0.95	14 (29.8)	87 (34.3)	0.34
Oral agents only	13 (24.1)	11 (20.4)		18 (33.3)	16 (29.6)		12 (25.5)	80 (31.5)	
Insulin only	16 (29.6)	16 (29.6)		11 (20.4)	10 (18.5)		10 (21.3)	53 (20.9)	
Oral agents and insulin	13 (24.1)	9 (16.7)		10 (18.5)	11 (20.4)		11 (23.4)	34 (13.4)	
PAD	8 (14.8)	9 (16.7)	0.79	7 (13.0)	8 (14.8)	0.78	6 (12.8)	25 (9.8)	0.55
Dyslipidemia	15 (27.8)	13 (24.1)	0.66	12 (22.2)	12 (22.2)	1.00	10 (21.3)	62 (24.4)	0.64
Hypertension	20 (37.0)	22 (40.7)	0.69	19 (35.2)	20 (37.0)	0.84	23 (48.9)	111 (43.7)	0.51
LVEF < 50%	11 (20.4)	8 (14.8)	0.45	9 (16.7)	10 (18.5)	0.80	11 (23.4)	27 (10.6)	**0.015**
Clinical Presentation									
ACS	35 (64.8)	37 (68.5)	0.68	34 (63.0)	36 (66.7)	0.69	30 (63.8)	137 (53.9)	0.21
SCAD	19 (35.2)	17 (31.5)		20 (37.0)	18 (33.3)		17 (36.2)	117 (46.1)	
Angiographic data									
Chronic total occlusion	7 (13.0)	7 (13.0)	1.00	7 (13.0)	9 (16.7)	0.59	9 (19.1)	40 (15.7)	0.56
Multivessel CAD	35 (64.8)	36 (66.7)	0.84	32 (59.3)	35 (64.8)	0.55	37 (78.7)	158 (62.2)	**0.029**
Calcification	8 (14.8)	10 (18.5)	0.61	7 (13.0)	8 (14.8)	0.78	9 (19.1)	36 (14.2)	0.38
ACC/AHA lesion B/C	36 (66.7)	36 (66.7)	1.00	37 (68.5)	35 (64.8)	0.68	30 (63.8)	165 (65.0)	0.88
SYNTAX score	22.9 ± 8.5	22.7 ± 9.3	0.91	24.6 ± 7.7	24.2 ± 9.0	0.81	21.7 ± 7.3	22.4 ± 8.5	0.63
Procedure data									
Stent diameter, mm	3.1 ± 0.4	3.0 ± 0.4	0.33	3.2 ± 0.4	3.1 ± 0.4	0.74	3.1 ± 0.5	3.1 ± 0.6	0.99
Stent length, mm	32.9 ± 17.4	34.3 ± 17.4	0.64	34.5 ± 14.7	31.7 ± 10.8	0.27	31.1 ± 14.5	35.0 ± 20.5	0.22
Complete revascularization	21 (38.9)	21 (38.9)	1.00	17 (31.5)	18 (33.3)	0.84	13 (27.7)	81 (31.9)	0.57
Stent type									
First generation DES	36 (66.7)	39 (72.2)	0.53	36 (66.7)	34 (63.0)	0.69	31 (66.0)	169 (66.5)	0.94
Second generation DES	18 (33.3)	15 (27.8)		18 (33.3)	20 (37.0)		16 (34.0)	85 (33.5)	

*MACEs* major adverse cardiovascular event; *BMI* body mass index; *FPG* fasting plasma glucose; *HbA1c* glycosylated haemoglobin; *PAD* peripheral artery disease; *LVEF* left ventricular ejection fraction; *ACS* acute coronary syndrome; *SCAD* stable coronary artery disease; *DES* drug-eluting stent

^a^Values are mean ± SD or n (%) as appropriate. *P* values are obtained from the student t test for continuous variables, and the Pearson χ^2^ test for categorical variables

The external validation set was a prospective study of 301 patients with T2DM who were treated with PCI at the Zhongnan Hospital of Wuhan University between May 2016 and Jun 2017. The exclusion criteria were the same as in the above nested case–control study. For all participants, clinical follow-up was performed at 30 days and 6, 9, 12 months after PCI, and angiographic follow-up was conducted at 12 months after PCI [17]. The primary outcome remained MACEs, while the secondary outcome was target lesion failure (TLF), a device-oriented composite endpoint of cardiac death, target vessel MI, and target lesion revascularization [18]. During a median follow-up of 12.4 months (IQR: 11.7–12.6 months), a total of 47 (15.6%) MACEs (all-cause death, n = 17; repeat revascularization, n = 22; MI, n = 9; stroke, n = 3) and 30 (10.0%) TLF were documented. This study followed the Transparent Reporting of a Multivariable Prediction Model for Individual Prognosis or Diagnosis (TRIPOD) statement [19]. More details in study design, baseline characteristics, and outcome definitions are summarized in Additional file 1: Supplementary Methods. The Study protocol was approved by local institutional review boards, and written informed consents were obtained from all participants.

### Untargeted metabolic profiling by liquid chromatography-mass spectrometry (LC–MS)

For all participants, serum samples were isolated from whole blood by centrifugation within 30 days before PCI, and stored at – 80 ℃ until use. To minimize the potential impacts of storage conditions on metabolite stability, we analyzed the level of methionine, a metabolite that could be extensively degraded if the frozen serum samples were stored too long or improperly [20]. As a result, the relative abundance of methionine for all samples was greater than 3 standard deviations below the mean (Additional file 1: Fig. S1), indicating that the serum samples were properly stored for metabolite detection.

Details in LC–MS procedures are described in Additional file 1: Supplementary Methods. Briefly, serum samples were first treated with acetonitrile/methanol (1:1, v/v) and isotope-labeled internal standard mixtures for metabolite extraction. Then, metabolite extracts were separated using an UPLC BEH Amide column (2.1 mm × 100 mm, 1.7 µm) on a Vanquish UHPLC system (Thermo, Waltham, USA). The column eluent was further detected for the acquisition of MS/MS spectra on a Q Exactive Orbitrap mass spectrometer (Thermo, Waltham, USA) operating in the positive and negative ion modes. The acquired MS raw data were analyzed on the XCMS Online platform (https://xcmsonline.scripps.edu) [21] for peak detection and metabolite annotation. The best-matched internal standard (B-MIS) normalization method [22] was used to normalize peak areas and yield relative abundance of metabolites. To ensure data quality, the quality control (QC) samples were prepared by mixing an equal aliquot of all samples, and run at the beginning of the sample queue for column conditioning and each of 10 samples thereafter. Metabolic peaks with relative standard deviations (RSD) of > 30% across QC samples or presenting in < 80% of QC samples were removed for further analysis [23].

### Targeted metabolite analysis in the external validation set

External validation of 6 metabolic biomarkers selected from untargeted metabolomic profiling was conducted by targeted metabolite analyses on a 20AD UPLC system (Shimadzu, Kyoto, Japan) coupled with a QTrap 5500 mass spectrometer (SCIEX, Framingham, USA) operating in the multiple reaction monitoring mode (Additional file 1: Supplementary Methods). The absolute concentration (µmol/L) of each metabolite in serum was determined using a 7-point calibration curve, created by calculating the peak area ratio of each calibrator (Sigma, St. Louis, USA) versus its concentration. All the calibration curves showed a good linearity, with R^2^ values of > 0.990, intra- and inter-batch precision values (as RSD) of < 15%, and accuracy values (as relative error) ranging from − 9.1 to 5.3% (Additional file 1: Table S1).

### In vitro experiments in HG-cultured HASMCs

HASMCs (LONZA) were grown in Dulbecco’s Modified Eagle’s medium (LONZA) supplemented with 25 mM D-glucose, 4 mM L-glutamine, 10% fetal bovine serum, and 1% penicillin/streptomycin in a humidified atmosphere at 37 ℃ and 5% CO_2_. After 3–5 passages, HG-cultured HASMCs were treated with 10 μM FK866 (an inhibitor of nicotinamide phosphoribosyltransferase, Sigma) for 20 h to block the salvage biosynthetic pathway of NAD^+^, or 200 μM 1-methyl-L-tryptophan (1MT, an inhibitor of indole-2,3-dioxygenase, Sigma) for 20 h to inhibit de novo synthesis of NAD^+^, or 10 μM β-nicotinamide mononucleotide (NMN, a NAD^+^ precursor, Sigma) for 20 h to sustain NAD^+^ levels [24]. For each group of HASMCs, NAD^+^ levels were detected by LC–MS; the activity of mitochondrial respiratory chain complexes (I–V) was measured using spectrophotometric assays [25]. The bioenergetic profile of HASMCs was determined by an XF24 Extracellular Flux Analyzer (Agilent, Santa Clara, USA) to calculate the parameters of mitochondrial respiration and glycolysis [26]. The mRNA expression and protein secretion of proinflammatory cytokines in HASMCs were examined by reverse transcription quantitative PCR and cytometric bead array (BD-Pharmingen), respectively. A transwell migration assay was performed to evaluate the ability of HASMCs to recruit THP1 monocytes [27]. Proliferation of HASMCs was assessed using a methylene blue dye assay, as described in our previous report [13]. All in vitro experiments were repeated 3 times. More Details are provided in Additional file 1: Supplementary Methods and Additional file 1: Table S2.

### Statistical analysis

In both discovery and internal validation sets, the global metabolic differences between participants with and without MACEs were assessed by a supervised model of orthogonal partial least-squares discriminate analysis (OPLS-DA). For assessing the robustness of OPLS-DA, we performed 200 permutations of the metabolomic datasets, and for each permutation, the values of Q^2^ and R^2^Y were calculated by a seven-fold cross validation to reflect the goodness of prediction and the risk of overfitting, respectively. The values of variable importance in the projection (VIP) were also calculated to reflect the contribution of each metabolite to the group discrimination in the OPLS-DA model. Differential comparisons of single metabolites between groups were examined using the Mann–Whitney U test, followed by the Benjamini–Hochberg FDR-controlling method [28] to adjust for multiple comparisons. Metabolites with VIP values of > 1.0, FDRs of < 0.05, and fold changes of > 4/3 or < 3/4 were considered as the differential metabolites [23, 29], which were further mapped into the KEGG database (https://www.kegg.jp/) for pathway enrichment analyses. Then, an optimal set of metabolic features for predicting MACEs was selected from the differential metabolites using the R package VSURF (Variable Selection Using Random Forests) [30], in which a recommended stepwise random forest (RF) procedure [31] was executed to identify the best combination of discriminant variables for classification prediction modeling on the basis of predictive accuracy (as the amount of out-of-bag error) and parsimony (as the number of selected variables).

In the discovery set, we integrated the metabolic features selected by VSURF to develop the reference model of logistic regression and 4 machine learning models for prediction of MACEs. The 4 machine learning algorithms were: (1) RF, an ensemble approach that produces multiple decision trees for classification [32], (2) extreme gradient boosting (XGBoost), another ensemble machine using the Shapley additive explanations method to create an additive model of decision trees [33], (3) nonlinear Support Vector Machines (SVM) with a polynomial kernel [34], and (4) deep multilayer neural network (DNN) with the adaptive moment estimation optimizer [35]. Details in model parameters are listed in the Additional file 1: Supplementary Methods. The importance of each feature to the prediction was assessed by Gini index in RF and by relative importance values in XGBoost. The predictive performance of 4 machine learning models was compared with that of the reference model (i.e., logistic regression) by measuring: (1) discrimination statistics including area under the receiver-operating characteristic curve (AUC), sensitivity, specificity, positive predictive value, and negative predictive value; (2) continuous net reclassification index (NRI); (3) calibration statistics (*P*-value of the Hosmer–Lemeshow test, calibration slope, and calibration plots); and (4) net clinical benefit through decision curve analyses [36]. To adjust for potential over-fitting and over-optimism, a seven-fold cross validation was also performed to obtain a bias-corrected AUC for each model.

We performed Kaplan–Meier curves and Cox regression to assess the prognostic values of the derived models. The proportional hazards assumption of Cox models was verified by visual inspection of log-minus-log plots and calculation of Schoenfeld residuals. Sensitivity analyses were also conducted to validate model performance after stratification by initial clinical presentations and different components of MACEs. For in vitro experiments, intergroup differences were compared using one-way ANOVA with LSD post hoc tests. All statistical analyses were conducted with R (version 3.5.3) and SIMCA-P (version 16.0.2). A *P*-value < 0.05 was considered significant.

## Results

### Baseline characteristics

As described in Table 1, after propensity score matching and random assignment, the baseline characteristics in both discovery and internal validation sets were similar between patients occurring MACEs (n = 54 for each set) and matched controls (n = 54 for each set). In the external validation set, MACEs (n = 47) were more likely to occur in patients who were current smokers or had left ventricular ejection fractions of < 50%, multivessel CAD, or higher levels of glycated hemoglobin at baseline (Table 1).

### Global metabolic alterations in patients with T2DM occurring MACEs after PCI

We first assessed the reliability of the LC–MS analysis using QC samples. As presented in Additional file 1: Figure S2, the Pearson correlation coefficients of metabolomic data among QC samples were greater than 0.99, indicating a good reproducibility of the LC–MS analysis. After data quality control and peak alignment, serum metabolome analyses annotated a total of 778 metabolites (discovery set: 743; internal validation set: 674; identified in both sets: 639), which were distributed across 10 ontology classes (Additional file 1: Table S3).

**Fig. 2:**
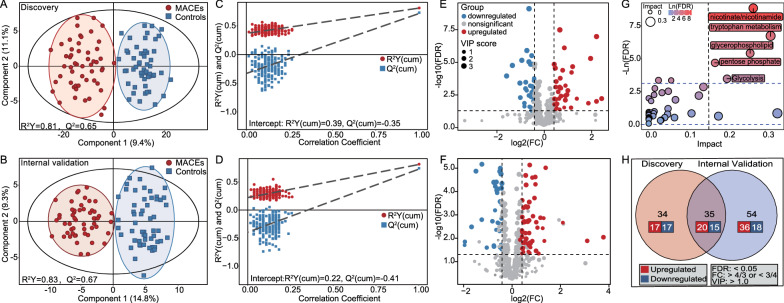
Differences in serum metabolome profiles between type 2 diabetic patients with and without the incidence of 1-year MACEs after PCI in the discovery and internal validation sets. **A** and **B** The OPLS-DA scatter plot. Each dot and square represents the serum metabolomic profile of a single participant in a 2-dimensional space. The ellipses represent 95% confidence intervals. Cross-validation plot with 200 permutations in the discovery (**C**) and internal validation sets (**D**). The volcano plot of differential metabolites in the discovery (**E**) and internal validation sets (**F**). The vertical dashed lines indicate the threshold of fold changes > 4/3 or < 3/4. The horizontal dashed line indicates the threshold of FDR < 0.05. **G** The pathway enrichment analysis of all differential metabolites. The horizontal dashed line indicates the threshold of FDR < 0.05. **H** The Venn diagram. *MACEs* major adverse cardiovascular events; *FDR* false discovery rate; *FC* fold change; *VIP* variable importance in the projection

As illustrated in the OPLS-DA models, the metabolomic profile of the MACEs group was significantly distinct from that of the matched control group in both discovery (R^2^Y = 0.81, Q^2^ = 0.65, Fig. 2A) and internal validation sets (R^2^Y = 0.83, Q^2^ = 0.67, Fig. 2B). After 200 permutations, the intercepts of goodness-of-prediction (Q^2^) and goodness-of-fit (R^2^) values were within ± 0.5 (Fig. 2C and D), indicating that the OPLS-DA models were well explained and not overfitting. Based on the conditions of VIP values > 1.0, FDR < 0.05, and fold changes > 4/3 or < 3/4, the volcano plots depicted 69 differential metabolites (37 upregulated, 32 downregulated, Fig. 2E and Additional file 1: Table S4) in the discovery set and 89 differential metabolites (56 upregulated, 33 downregulated, Fig. 2F and Additional file 1: Table S5) in the internal validation set. Pathway enrichment analyses of the differential metabolites found 4 metabolic pathways significantly perturbed in patients with incident MACEs, involving nicotinate and nicotinamide metabolism, tryptophan metabolism, glycerophospholipid metabolism, pentose phosphate pathway, and glycolysis (Fig. 2G). Of all the differential metabolites, 35 were identified in both discovery and internal validation sets (20 upregulated, 15 downregulated, Fig. 2H), with the potential to better distinguish MACEs from matched controls (Additional file 1: Figure S3).

### Development and internal validation of a 6-metbolite signature to predict MACEs

**Fig. 3:**
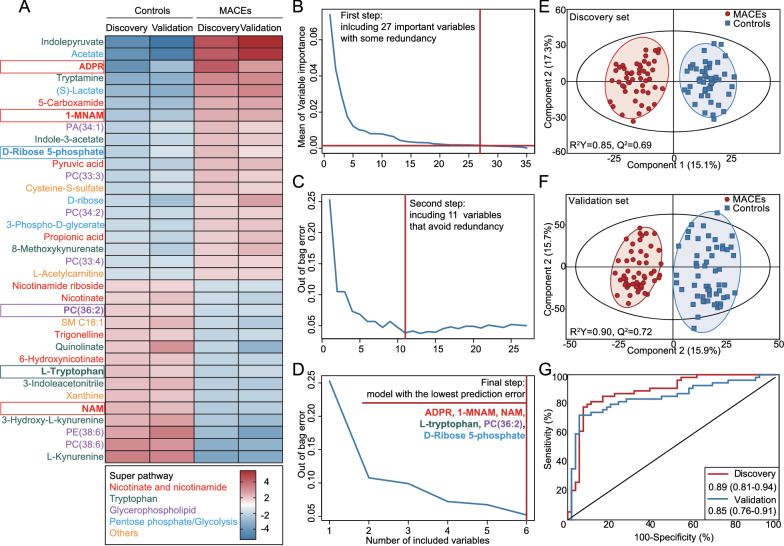
The variable selection process for identifying a 6-metabolite signature to predict 1-year MACEs after PCI among patients with T2DM. **A** Hierarchical cluster analysis of the 35 metabolites differentially expressed in both discovery and internal validation sets. **B** Of the 35 metabolites, 27 important metabolites with some redundancy are selected in the first step of a variable selection algorithm (VSURF). **C** Of the remaining 27 metabolites, 11 metabolites that avoid redundancy are further selected in the second step of VSURF. **D** The final step of VSURF identifies a combination of 6 metabolites with the best potential for predicting 1-year MACEs. The OPLS-DA scatter plot based on the 6 metabolites in both discovery (**E**) and internal validation (**F**) sets. **G** The ROC analysis of the 6 metabolites based on logistic regression. *ADPR* adenosine diphosphate ribose; *1-MNAM* 1-Methylnicotinamide; *PC* phosphatidylcholine; *NAM* nicotinamide

From the 35 metabolites differentially expressed in both data sets (Fig. 3A), we sought to select an optimal set of discriminators for MACEs by considering the balance between classification accuracy and parsimony. For this purpose, the normalized data of the 35 metabolites from the discovery set were inputted into a tree-based VSURF algorithm, in which a total of 29 metabolites were gradually excluded due to low importance or high redundancy (Fig. 3B and C), finally leaving a subset of 6 metabolites with the lowest prediction error for multivariate modeling (Fig. 3D). Of particular note, among the 6 metabolites, 4 were involved in biosynthesis (nicotinamide [NAM] and L-tryptophan), consumption (adenosine diphosphate ribose [ADPR]), or excretion [1-methylnicotinamide (1-MNAM)] of NAD^+^ [37], implying a crucial role of NAD^+^ metabolism in the occurrence of MACEs among diabetic patients who received PCI.

As depicted in the OPLS-DA models (Fig. 3E and F), the combination of these 6 metabolites could clearly separated patients with MACEs from matched controls in both discovery and internal validation sets. The logistic regression model composed of these 6 metabolites yielded an AUC of 0.89 [95% confidence interval (CI) 0.81–0.94] in the discovery set and 0.85 (95% CI 0.76–0.91) in the internal validation set for predicting MACEs (Fig. 3G). When the 2 datasets were divided into high-(> 62%) and low-risk (≤ 62%) groups based on the risk probability predicted by the 6-metabolite model (cut-off value derived from the ROC analysis), Kaplan–Meier estimates of the rates of MACEs significantly differed between high- and low-risk groups (*P* < 0.001, Additional file 1: Figure S4). After multivariable adjustment by potential confounding factors, the 6-metabolite model remained a powerful and independent prognostic predictor for MACEs [discovery set: hazard ratio (HR) = 8.92; internal validation set: HR = 6.17, both *P* < 0.001, Additional file 1: Figure S4].

### Improving performance of the 6-metabolite model using machine learning

To evaluate whether the predictive performance of the 6-metabolite panel could be improved by the application of machine learning, we developed the 6-metabolite prediction models by incorporating the data of the discovery set into 4 machine learning algorithms: RF, XGBoost, SVM, and DNN. As summarized in Table 2, all of the machine learning models, except for DNN, yielded a significantly higher AUC (0.93–0.99, *P*_*difference*_ < 0.05) than the reference model of logistic regression (AUC = 0.89). Likewise, the reclassification ability of 4 machine learning models was also improved, with continuous NRIs ranging from 0.96 to 1.93.

**Table 2:** Discrimination and reclassification ability of the 6-metabolite model based on 4 machine learning algorithms and logistic regression

Model	Discrimination statistics						Seven-fold CV	Reclassification index	
	AUC	*P* value^a^	Sensitivity	Specificity	PPV	NPV	AUC	NRI	*P* value
Discovery set									
Logistic regression	0.89 (0.81–0.94)	Ref.	80 (67–89)	91 (80–97)	61 (40–79)	96 (94–98)	0.89	Ref.	Ref.
Random forest	0.99 (0.95–1.00)	< 0.001	98 (90–99)	96 (87–99)	83 (55–95)	99 (98–100)	0.99	1.93 (1.83–2.03)	< 0.001
XGBoost	0.99 (0.96–1.00)	< 0.001	98 (90–99)	96 (87–99)	83 (55–95)	99 (98–100)	0.99	1.89 (1.77–2.01)	< 0.001
SVM	0.93 (0.86–0.97)	0.005	85 (73–93)	93 (82–98)	68 (45–84)	97 (95–99)	0.91	1.00 (0.68–1.32)	< 0.001
DNN	0.91 (0.85–0.96)	0.092	96 (87–99)	87 (75–95)	58 (40–73)	99 (97–100)	0.92	0.96 (0.66–1.26)	< 0.001
Internal validation									
Logistic regression	0.85 (0.76–0.91)	Ref.	72 (58–84)	94 (85–99)	70 (44–88)	95 (92–97)	0.85	Ref.	Ref.
Random forest	0.93 (0.87–0.97)	0.003	83 (71–92)	94 (85–99)	73 (48–89)	97 (95–98)	0.91	1.33 (1.05–1.61)	< 0.001
XGBoost	0.91 (0.84–0.95)	0.023	85 (73–93)	94 (85–99)	73 (48–89)	97 (95–99)	0.91	1.33 (1.05–1.61)	< 0.001
SVM	0.89 (0.82–0.94)	0.119	85 (73–93)	83 (71–92)	48 (34–63)	97 (94–98)	0.88	0.22 (− 0.15 to 0.59)	0.240
DNN	0.82 (0.74–0.89)	0.329	69 (54–81)	94 (85–99)	69 (42–87)	94 (92–96)	0.84	0.22 (− 0.14 to 0.59)	0.233

*AUC* area under the curve; *PPV* positive predictive value; *NPV* negative predictive value; *CV* cross validation; *NRI* net reclassification improvement; *XGBoost* extreme gradient boosting tree; *SVM* Support Vector Machines; *DNN* deep neural network

^a^Paired comparisons of AUCs between logistic regression and each machine learning model are conducted using a DeLong test

When the machine learning models were applied in the internal validation set, the top 2 best-performing models were RF and XGBoost models, which both showed significant improvements in discrimination and reclassification abilities to predict MACEs compared with the logistical regression model. Specifically, sensitivities for the RF and XGBoost models increased to  ~ 85% compared with 72% for the logistic regression model, meaning that about 13% (7/54) of patients who developed MACEs after PCI would be correctly identified using the RF and XGBoost models but would be missed when the logistic regression model was applied (Additional file 1: Figure S5). Interestingly, all 4 metabolites related to NAD^+^ metabolism were highlighted as the top 4 important variables to the predictive outcomes in both RF and XGBoost models (Additional file 1: Figure S6). Otherwise, the SVM and DNN models did not improve predictive performance relative to the regression model in the internal validation set.

For all prediction models, the average AUCs calculated by cross validation remained largely unchanged (Table 2); calibration plots showed a good agreement between predicted and observed outcomes (calibration slope around 1, Additional file 1: Figure S7).

### External validation using a targeted metabolite analysis

**Fig. 4:**
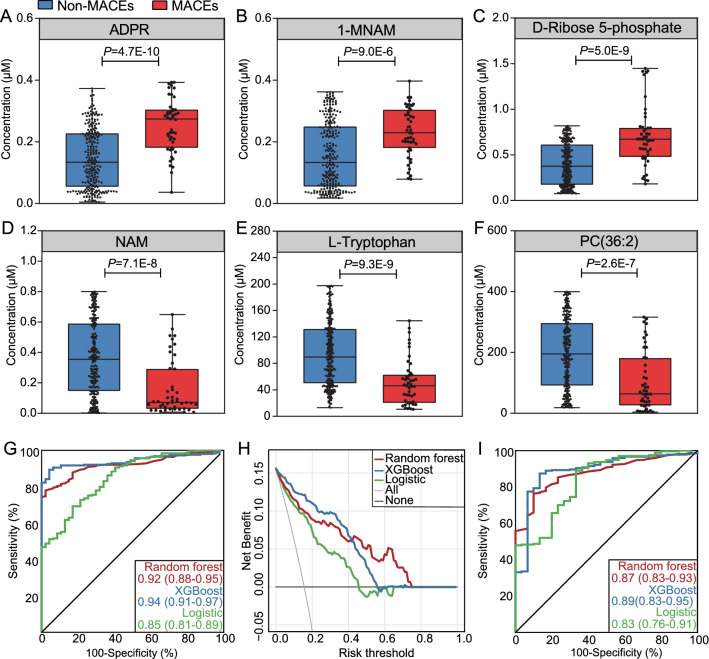
External validation of the 6-metabolite signature by targeted metabolite analyses in a prospective cohort of 301 type 2 diabetic patients undergoing PCI. Comparisons of the absolute concentrations of ADPR (**A**), 1-MNAM (**B**), D-Ribose 5-phosphate (**C**), NAM (**D**), L-Tryptophan (**E**), and PC(36:2) (**F**) between patients with and without the incidence of MACEs at 1-year after PCI. **G** The improvements in AUCs of the 6-metabolite models based on random forest or XGBoost compared with the logistic regression model. **H** The greater clinical benefit of 6-metabolite model based on random forest or XGBoost compared with the logistic regression model. **I** The ROC analysis of the 6-metabolite models for predicting target lesion failure at 1 year after PCI. *ADPR* adenosine diphosphate ribose; *1-MNAM* 1-Methylnicotinamide; *PC* phosphatidylcholine; *NAM* nicotinamide; *XGBoost* gradient-boosted decision tree

When determining the absolute concentrations of the 6 metabolites using a targeted metabolite analysis in the external validation cohort, we found that 3 of the 6 metabolites were associated with a higher risk of MACEs and the other 3 were associated with a lower risk of MACEs (Fig. 4A–F), which were consistent with the results from the untargeted metabolomics mentioned above. After inputting the normalized (also used the B-MIS method) data of the 6 metabolites into the established machining learning models, the AUCs for predicting MACEs reached to 0.92 (95% CI 0.88–0.95, *P*_*difference*_ = 0.005, Fig. 4G) in RF and 0.94 (95% CI 0.91–0.97, *P*_*difference*_ < 0.001, Fig. 4G) in XGBoost, compared to 0.85 (95% CI 0.81–89) in logistic regression. Decision curve analyses also showed that both RF and XGBoost models had larger net benefits (i.e., a greater number of appropriate triage) across the range of risk thresholds compared with the logistic regression model (Fig. 4H). When categorizing participants into high-risk and low-risk groups based on the prediction of the 6-metbaolite classifier, the adjusted HR for MACEs was 7.68 (95% CI 3.57–16.55, Additional file 1: Figure S8) for the comparison of high-risk versus low-risk groups. Likewise, the 6-metabolite classifier achieved a smaller but still good AUC (0.83–0.89) to predict the device-oriented endpoint of TLF (Fig. 4I).

Recently, the FREEDOM trial derived a personalized clinical risk model for MACE prediction in diabetic patients undergoing revascularization [38]. Here, we further assessed the additional value of our 6-metabolite model beyond the FREEDOM tool. As shown in Additional file 1: Table S6, adding the RF-based 6-metbolite model into the FREEDOM tool substantially increased the C-index to 0.87 (95% CI 0.81–0.93) for predicting MACEs. The classification performance was also improved after addition of the 6-metabolite panel, with a categorical NRI of 0.60 (95% CI 0.45–0.75, *P* < 0.001) and an IDI of 0.27 (95% CI 0.22–0.32, *P* < 0.001).

### Internal and external validation by sensitivity analyses

**Fig. 5:**
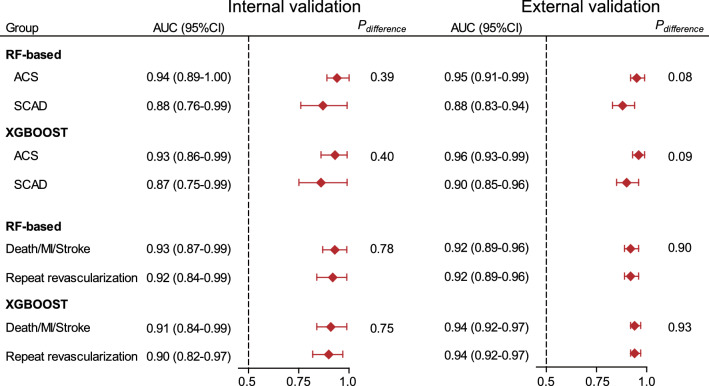
Sensitivity analyses for internally and externally validating the predictive performance of the 6-metabolite model after stratification by initial clinical presentations and different components of MACEs. *RF* random forest; *XGBoost* gradient-boosted decision tree; *ACS* acute coronary syndrome; *SCAD* stable coronary artery disease; *MI* myocardial infarction

We first performed sensitivity analyses for assessing the performance of the 6-metabolite model in predicting different components of MACEs. As shown in Fig. 5, the 6-metabolite model consistently yielded high AUCs (≥ 0.90) for either predicting the combined end point of death, MI, and stroke or predicting repeat revascularization in both internal and external validation sets.

Then, considering that the prognosis following PCI significantly differed between patients initially presenting with acute coronary artery syndrome (ACS) and stable CAD (SCAD) [39], sensitivity analyses were further conducted after stratification by initial clinical presentations. We observed that the AUCs of the 6-metabolite model for predicting MACEs were generally higher (0.93–0.96) in patients presenting with ACS, and slightly lowered to 0.87–0.90 in patients presenting with SCAD, but the differences were not statistically significant (Fig. 5).

### Effects of altering NAD^+^ biosynthesis in HG-cultured HASMCs

**Fig. 6:**
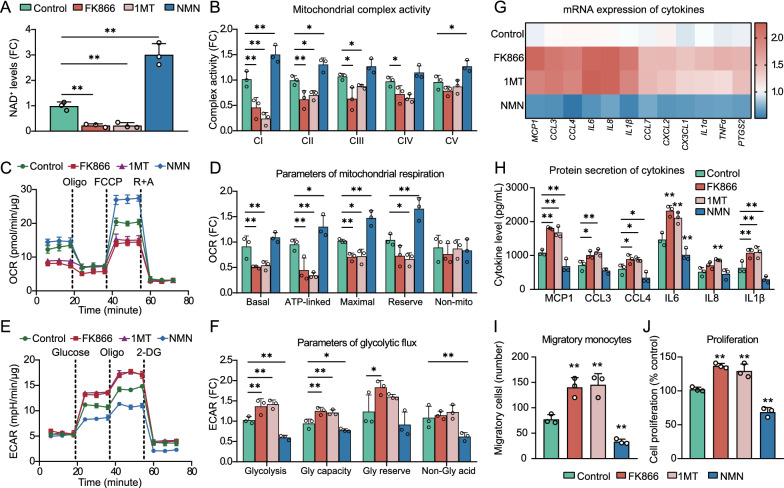
Effects of inhibiting (FK866 or 1MT) or sustaining (by NMN) NAD^+^ biosynthesis on bioenergetic profiles, inflammatory activation, and proliferation of HASMCs under high glucose conditions. **A** NAD^+^ levels detected by LC–MS. **B** Mitochondrial complex activity. **C** Mitochondrial stress test where different parameters of mitochondrial respiration are compartmentalized by a sequential application of Oligo, FCCP, and a combination of rotenone and antimycin. **D** Parameters of mitochondrial respiration. **E** Glycolysis stress test where different parameters of glycolytic flux are compartmentalized by a sequential application of glucose, Oligo, and 2DG. **F** Parameters of glycolytic flux. **G** Heatmap of mRNA expression of inflammatory cytokines determined by RT-qPCR. **H** Protein secretion of inflammatory cytokines. **I** Number of THP1 monocytes migrating toward HASMCs. **J** Proliferation of HASMCs. For all in vitro experiments, the control group is HG-cultured HASMCs. Data are presented as mean ± SD, n = 3 independent experiments. *P < 0.05; **P < 0.01. *1MT* 1-methyl-L-tryptophan; *NMN* nicotinamide mononucleotide; *Oligo* oligomycin; *R* rotenone; *A* antimycin; *OCR* oxygen consumption rate; *2DG* 2-deoxy-glucose; *ECAR* extracellular acidification rate; *FC* fold change; *Gly* glycolytic

Considering the importance of NAD^+^ metabolites in gaining predictive performance of our prediction model, we investigated the effects of altering NAD^+^ biosynthesis on the phenotypes of HASMCs under HG conditions. As shown in Fig. 6A and B, pharmacological inhibition of NAD^+^ biosynthesis by FK866 or 1MT led to a substantial reduction in basal NAD^+^ levels, accompanied by a marked deficit in mitochondrial complex I activity, which requires reduced NAD^+^ for mitochondrial electron transfer [40]. Consistent with abnormal changes in activities of mitochondrial complexes, significant reductions in parameters of mitochondrial respiratory, including mitochondrial basal respiration, ATP-linked respiratory capacity, and maximal respiration (Fig. 6C and D), were observed along with increases in glycolytic flux after pharmacological blockade of NAD^+^ biosynthesis in HASMCs (Fig. 6E and F). Conversely, supplementation of NMN, a NAD^+^ precursor, increased basal NAD^+^ levels and enhanced mitochondrial respiratory capacities while decreasing glycolysis in HG-cultured HASMCs (Fig. 6A–F).

Given the potential link of aerobic glycolysis to inflammatory activation [41], we elected to further explore the impact of interfering NAD^+^ biosynthesis on the HG-induced expression of an array of proinflammatory factors with documented roles in cardiovascular disease. As a result, inhibition of NAD^+^ biosynthesis by FK866 or 1MT in HASMCs was found to significantly increase the production of a series of chemokines (MCP1, CCL3, CCL4, etc.) and interleukins (IL6, IL8, IL-1β etc.), both in terms of mRNA expression and protein secretion (Fig. 6G and H). In parallel, exposure of HASMCs to FK866 or 1MT resulted in a more than 80% increase in chemotaxis of THP1 monocytes toward HASMCs (Fig. 6I), along with increased proliferation of HASMCs (Fig. 6J). In contrast, NMN supplementation normalized the production of proinflammatory factors, attenuated the ability of HASMCs to recruit THP1 monocytes, and inhibited HASMCs proliferation (Fig. 6G–J).

## Discussion

Diabetes is deemed as one of the most significant prognostic factors for adverse outcomes following PCI, with hazard ratios ranging from 1.9 to 2.5 [42]. Mounting evidence also indicates that diabetes causes increased proliferation of vascular smooth muscle cells [43], more extensive neointimal hyperplasia [44], and consequent more severe restenosis after stent implantation [45], highlighting the view that the mechanism underlying the adverse outcomes after PCI in diabetes is probably different from that in non-diabetes [42]. Specific to metabolomic studies, there has been epidemiological evidence showing that diabetic patients with macrovascular complications have distinct circulating metabolic profiles compared with those without [46, 47]. Recently, Cui and colleagues constructed a metabolite panel of phospholipids and sphingolipids with high accuracy (AUC > 0.90) for diagnosis of stent restenosis in non-diabetic patients [48]. However, this metabolite panel only achieved a modest AUC of < 0.70 (data not shown) for predicting MACEs in our cohorts of type 2 diabetic patients receiving PCI, implying that the metabolite fingerprint causally associated with the occurrence of adverse outcomes after PCI may also differ between diabetic and non-diabetic status.

Hence, for the first time, the present study focused on the identification of differential metabolic patterns at baseline to predict the incidence of MACEs at 1 year after PCI for patients with T2DM. By applying both untargeted and targeted metabolomic approaches, we first found a significant difference in serum metabolome profiles between type 2 diabetic patients with and without incident MACEs following PCI. Then, the Venn diagram depicted 35 metabolites differentially expressed in both discovery and internal validation sets, which had the potential to better discriminate patients with the occurrence of MACEs from matched controls. For constructing the parsimonious model that would be more achievable in the clinical setting, our next step was to select an optimal set of predictors from the 35 metabolites.

Considering that the 35 metabolites belong to several metabolic pathways with possible interconnections, we did not use the traditional feature selection methods like logistic regression, because fitting a logistic regression model is, in fact, algebraically difficult when there are too many variables with complex interactions between each other [11]. Instead, the 35 metabolic features were filtered by the VSURF algorithm, which is a recommended tree-based method to identify the most important variables for classification after accounting for the complex, nonlinear relations in the dataset [11, 31]. As a result, a total of 6 metabolic features, including 4 NAD^+^ metabolites, 1 phosphatidylcholine lipid, and 1 sugar phosphate, were finally selected for multivariate modeling.

Then, we went on to consider how the 6 metabolites could best be incorporated to increase predictive accuracy. For this purpose, we trained the 6 metabolites using a series of powerful machine learning algorithms, including ensemble methods like RF and XGBoost, nonlinear method like SVM, and multilayer neural network. The testing results showed that the 6-metbolite models based on RF and XGBoost were the best-performing models, with high discriminative accuracy of ≥ 90% in both internal and external validation cohorts. Of note, the two ensemble models could detect an additional 13% of patients in whom the occurrence of MACEs following PCI would not be identified when using the traditional logistic regression model. If these findings can be verified, our 6-metabolite classifier may be as a helpful tool for identifying patients at high risk for MACEs and guiding early prevention among patients with T2DM undergoing PCI.

An interesting finding of the current study is that our prediction model includes 4 key metabolites involved in biosynthesis (NAM, L-tryptophan), consumption (ADPR), or secretion (1-MNAM) of NAD^+^, suggesting a possible link of NAD^+^ metabolism to adverse outcomes following PCI. Physiologically, NAD^+^ can be synthesized de novo starting with tryptophan, or from salvage pathway starting with NAD^+^ precursors like NAM derived from cellular NAD^+^ metabolism or dietary supply [49]. Under critical conditions (e.g., acute DNA injury and cell death), the synthesized NAD^+^ can be excessively consumed by hyperactivated poly(ADP-ribose) polymerases to produce ADPR and NAM as byproducts, in which ADPR continues to form poly(ADP-ribose) chains with pivotal effects on posttranslational modification of target proteins [50], whereas NAM can be methylated to form MNAM that is mainly secreted via urine [37]. Our previous bidirectional Mendelian Randomization study has shown that higher extent of leukocyte poly(ADP-ribose), as a hallmark of massive NAD^+^ consumption, is causally associated with the incidence of 1-year MACEs after PCI [12]. The present study extends our previous work by providing experimental evidence that pharmacologically blocking the salvage or de novo biosynthetic pathways of NAD^+^ causes abnormal changes in bioenergetic profiles, upregulated expression of proinflammatory factors, increased chemotaxis of monocytes to HASMCs, and enhanced proliferation of HASMCs. In contrast, sustaining NAD^+^ levels via NMN supplementation may inhibit HG-induced glycolysis, pro-inflammation, and proliferation of HASMCs, all of which are aberrant phenotypes related to incident MACEs after PCI [42, 51]. These findings are in line with recent evidence that the intrinsic NAD^+^ fueling system is essential to protect against DNA damage, premature senescence, and chaotic migration of smooth muscle cells [52, 53], supporting the close link between NAD^+^ biosynthesis and phenotypic switching of HASMCs. Collectively, our in vitro data may provide an experimental foundation for the significant effects of NAD^+^ metabolites on post-PCI outcomes.

Our study is the first to develop a machine learning-based metabolite classifier to predict incident MACEs at 1 year after PCI for type 2 diabetic patients, with rigorous steps for model specification and evaluation of model performance (i.e., discrimination, calibration, and clinical usefulness). Nevertheless, our study also has limitations. First, owing to a relatively short follow-up period, we could not evaluate the predictive performance of our 6-metabolite model for long-term outcomes after PCI. Second, although we validated the 6-metabolite model using both internal and external datasets, its predictive utility should be extended in larger independent cohorts, especially in other geographic populations. Third, sensitivity analyses observed that the predictive accuracy of the 6-metabolite model might slightly decrease in patients initially presenting with SCAD. So we could not rule out the possibility that simultaneously enrolling patients with different clinical presentations might potentially confound the performance of our prediction model. Fourth, other angiographic parameters, such as lesion type, vessel tortuosity, and presence of thrombus, might provide additional information on disease progression, but were not available in the current study. Fifth, although we computed variable importance to define predictors that most affected classification, the prediction model based on RF and XGBoost might still be harder to be interpreted, compared with the regression model simply using given coefficients to weight predictors [54]. Finally, more experimental research is needed for better understanding of the exact mechanism underlying the predictive value of NAD^+^ metabolites.

## Conclusions

Using an array of machine learning algorithms, we develop a 6-metabolite signature with high accuracy for predicting incident MACEs at 1 year after PCI in patients with T2DM. A diagnostic test based on this metabolite model is clinically achievable because of the small number of metabolites included in our model, and may have a potential utility of early identification of type 2 diabetic patients at high risk for post-PCI outcomes. Our study also provides the first evidence for a critical role of abnormal changes in NAD^+^ metabolites in the occurrence of adverse outcomes after PCI under diabetic conditions.

## Supplementary Information

**Additional file 1: Supplementary methods.** **Figure S1.** The Z distribution of methionine abundance in both discovery and internal validation sets. The Z scores of methionine across all samples were greater than -3 (-3SD), indicating that the serum samples were properly stored for metabolite detection. **Figure S2.** The Pearson correlation between metabolic data of quality control samples assessing the reliability of the LC-MS analysis in the discovery (**A**) and internal validation sets (**B**). **Figure S3.** The OPLS-DA analysis assessing the performance of 35 differential metabolites for discrimination of MACEs from matched controls in the discovery (**A**) and internal validation sets (**B**). **Figure S4. **The differences in cumulative rates of MACEs between participants with high-risk and low risk scores of the 6-metabolite signatures. *P* values were derived from Cox regression with adjustment for age, sex, smoking status, obesity (BMI > 25 kg/m^2^), hypertension, HbA1c, LVEF < 50%, clinical presentations, multivessel CAD, SYNTAX score, and stent types. **Figure S5.** Scatter plots for comparing the predictive performance of the random forest (**A**) and XGBoost (**B**) models to the logistic regression model of the 6-metabolite panel. The models are generated using the discovery dataset and presented here in the internal validation set. Red lines indicate the cut-offs of random forest and XGBoost models; Black lines indicate the cut-off of logistic regression model. Black circles label MACEs that would be identified using the random forest and XGBoost models but would be missed when the logistic regression model is applied. **Figure S6.** The importance of each predictor of the 6-metabolite classifier constructed by random forest (**A**) and XGBoost (**B**). Abbreviations: NAM, nicotinamide; ADPR, adenosine diphosphate ribose; 1-MNAM, 1-methylnicotinamide; PC, phosphatidylcholine. **Figure S7.** Calibration plots for the logistic regression and 4 machine learning models of the 6-metabolite classifier in the discovery (**A**) and internal validation sets (**B**). Abbreviations: RF, random forest; XGBoost, extreme gradient boosting; SVM, Support Vector Machines; DNN, deep neural network. **Figure S8.** Backward stepwise Cox regression analyses of the association between the 6-metabolite classifier and MACEs in the external validation set. (A) The log-minus-log plot for graphically testing the proportional hazards assumption. (B) Kaplan-Meier curve for assessing the performance of the 6-metabolite classifier to predict MACEs. In the backward stepwise Cox regression analyses, variables including age, sex, smoking status, obesity (BMI > 25 kg/m^2^), hypertension, HbA1c, LVEF < 50%, clinical presentations, multivessel CAD, SYNTAX score, stent types, and the 6-metabolite classifier were first entered one at a time. Then, 4 variables with *P*< 0.10 (i.e. HbA1c, LVEF < 50%, multivessel CAD, and the 6-metabolite classifier) in the stepwise procedure were retained to fit the final model. The HR and P value were calculated accordingly. **Table S1.** Calibration data for 6 metabolites detected by targeted metabolite analyses. **Table S2.** List of primers used in RT-qPCR. **Table S3.** Ontology classes of metabolites in the discovery and internal validation sets. **Table S4.** 69 differential metabolites in the discovery set. **Table S5.** 89 differential metabolites in the internal validation set. **Table S6.** The additional values of the RF-based 6-metabolite model beyond the FREEDOM clinical risk score in the external validation set.

## Data Availability

All data and methods supporting the findings of this study are available from the corresponding author upon reasonable request.

## Abbreviations

T2DM: Type 2 diabetes mellitus
CAD: Coronary artery disease
PCI: Percutaneous coronary intervention
MACEs: Major adverse cardiovascular events
NAD: Nicotinamide adenine dinucleotide
HASMCs: Human aortic smooth muscle cells
HG: High glucose
FDR: False discovery rate
TLF: Target lesion failure
LC–MS: Liquid chromatography-mass spectrometry
1MT: 1-Methyl-L-tryptophan
NMN: Nicotinamide mononucleotide
OPLS-DA: Orthogonal partial least-squares discriminate analysis
VIP: Variable importance in the projection
VSURF: Variable selection using random forests
RF: Random forest
XGBoost: Extreme gradient boosting
SVM: Support vector machines
DNN: Deep neural network
AUC: Area under the receiver-operating characteristic curve
NAM: Nicotinamide
ADPR: Adenosine diphosphate ribose
1-MNAM: 1-Methylnicotinamide
ACS: Acute coronary syndrome
