# Supplementary material for: A novel 6-metabolite signature for prediction of clinical outcomes in type 2 diabetic patients undergoing percutaneous coronary intervention
Source: Cardiovasc Diabetol. 2022 Jul 4;21:126. doi: 10.1186/s12933-022-01561-1 (PMC9254602; doi:10.1186/s12933-022-01561-1)
Supplement: Supplementary file 1 — Additional file 1: Supplementary methods. Figure S1. The Z distribution of methionine abundance in both discovery and internal validation sets. The Z scores of methionine across all samples were greater than -3 (-3SD), indicating that the serum samples were properly stored for metabolite detection. Figure S2. The Pearson correlation between metabolic data of quality control samples assessing the reliability of the LC-MS analysis in the discovery (A) and internal validation sets (B). Figure S3. The OPLS-DA analysis assessing the performance of 35 differential metabolites for discrimination of MACEs from matched controls in the discovery (A) and internal validation sets (B). Figure S4. The differences in cumulative rates of MACEs between participants with high-risk and low risk scores of the 6-metabolite signatures. P values were derived from Cox regression with adjustment for age, sex, smoking status, obesity (BMI > 25 kg/m2), hypertension, HbA1c, LVEF < 50%, clinical presentations, multivessel CAD, SYNTAX score, and stent types. Figure S5. Scatter plots for comparing the predictive performance of the random forest (A) and XGBoost (B) models to the logistic regression model of the 6-metabolite panel. The models are generated using the discovery dataset and presented here in the internal validation set. Red lines indicate the cut-offs of random forest and XGBoost models; Black lines indicate the cut-off of logistic regression model. Black circles label MACEs that would be identified using the random forest and XGBoost models but would be missed when the logistic regression model is applied. Figure S6. The importance of each predictor of the 6-metabolite classifier constructed by random forest (A) and XGBoost (B). Abbreviations: NAM, nicotinamide; ADPR, adenosine diphosphate ribose; 1-MNAM, 1-methylnicotinamide; PC, phosphatidylcholine. Figure S7. Calibration plots for the logistic regression and 4 machine learning models of the 6-metabolite classifier in the discovery [file 12933_2022_1561_MOESM1_ESM.pdf]

SUPPLEMENTAL MATERIALS

A novel 6-metabolite signature for prediction of clinical outcomes in type 2 diabetic patients undergoing percutaneous coronary intervention

Correspondence: Xue-bin Wang

Department of Clinical Laboratory, Key Clinical Laboratory of Henan Province, The First Affiliated Hospital of Zhengzhou University, Jianshe East Road No.1, Zhengzhou 450000, Henan, China. E-mail: xbwang2017@163.com

The Supplementary Materials includes:

Supplementary Methods

Supplementary Figures S1 to S8

Supplementary Tables S1 to S6

Catalogue

Supplementary Methods **3**

1. Baseline and outcome data 3
2. Untargeted metabolomic profiling 5
3. Targeted metabolite analysis 6
4. Machine learning algorithms 8
5. In vitro experiments 9

Supplementary Figures **13**

Figure S1. 13

Figure S2. 13

Figure S3. 14

Figure S4. 14

Figure S5. 15

Figure S6 15

Figure S7. 16

Figure S8. 16

Supplementary Tables **17**

Table S1 17

Table S2 18

Table S3 18

Table S4 19

Table S5 21

Table S6 24

References **25**

Supplementary Methods

1. Baseline and outcome data

1.1 Diagnosis of type 2 diabetes mellitus (T2DM)

T2DM was diagnosed based on the 2014 criteria of the American Diabetes Association: fasting plasma glucose ≥ 7.0 mmol/L or 2-h postchallenge plasma glucose ≥ 11.1 mmol/L or glycated hemoglobin ≥ 6.5% or ongoing therapy for T2DM [1].

1.2 Definition of clinical characteristics

Current smokers were defined as having smoked ≥ 100 cigarettes in their lifetime and now smoking every day or some days. Hypertension was defined as ongoing therapy for hypertension, systolic blood pressure of ≥ 140mmHg or diastolic blood pressure of ≥ 90mmHg. Dyslipidemia was defined as hypercholesterolemia (serum total cholesterol > 5.72 mmol/L), high levels of low-density lipoprotein cholesterol (> 3.1 mmol/L), low levels of high-density lipoprotein cholesterol (< 0.9 mmol/L), and hypertriglyceridemia (serum triglyceride > 1.70 mmol/L). Peripheral vascular disease was defined as arteries other than coronaries, with exercise-related claudication, revascularization surgery, reduced or absent pulsation, and/or angiographic stenosis of > 50%. Left ventricular end-systolic and end-diastolic volumes were measured using a standard ultrasound machine with a 2.5-MHz probe, and left ventricular ejection fraction was calculated by Simpson biplane method [2].

1.3 Angiographic characteristics

Before primary percutaneous coronary intervention (PCI), all participants

underwent invasive coronary angiography using the Judkins percutaneous trans-femoral technique. Digital angiograms were reviewed by two expert observers to document lesion characteristics. From the angiograms, moderate (readily visible but mild degree) or severe (obvious, heavy degree) calcification was recorded [3]. Chronic total occlusion was defined as a luminal occlusion in a native coronary artery with no or minimal contrast penetration through the lesion. Multivessel coronary artery disease (CAD) was defined as coronary artery stenosis of ≥ 50% in at least 2 major epicardial coronary arteries. The anatomical complexity of CAD was assessed using the SYNTAX score [4]. Interobserver variability between 2 observers was 0.84 (0.77–0.91), and intraobserver variability was 0.96 (0.93–0.98).

1.4 Study outcome

The primary outcome for all datasets was major adverse cardiovascular events (MACEs), a patient-oriented composite endpoint composed of all-cause death, myocardial infarction (MI), stroke, and repeat revascularization. All-cause death was defined as death from any cause. MI was defined as ischaemic signs or symptoms and new pathological Q-waves in ≥ 2 contiguous ECG leads, or/and an elevation CK-MB or troponin above the 99th percentile limit of normal and at least ≥ 20% above the most recent value. Stroke was defined as a focal neurologic deficit of central origin lasting > 72 h, or a focal neurologic deficit of central origin lasting > 24 h, with imaging evidence of cerebral infarction or intracerebral hemorrhage. Repeat revascularization was defined as any repeat PCI. All stages of a staged index PCI procedure would be considered part of the index revascularization procedure and not a

repeated revascularization.

The secondary outcome of the external validation set was target lesion failure, a device-oriented composite endpoint of cardiac death, target vessel-MI, and target lesion revascularization. Cardiac death was defined as any death due to proximate cardiac cause (MI, significant cardiac arrhythmia, refractory congestive heart failure, etc), procedure-related death, and death of unclear cause [5]. Target lesion revascularization was defined as any repeat PCI of the target lesion as a result of restenosis or other complications of the target lesion.

2. Untargeted metabolomic profiling

2.1 Serum sample preparation

At first, 180 μL of serum samples was mixed with 800 μL of acetonitrile/methanol (1: 1, v/v) and 20 μL of internal standard (L-2-Chlorophenylalanine, 2 μg/mL). Then, the mixture was vortexed for 30 s, sonicated for 10 min, and incubated for 1 h at -20 ℃ to precipitate proteins. After centrifugation at 13,000 *g* for 15 min at 4 ℃, the supernatant (800 μL) of the mixture was carefully collected, dried under nitrogen, and resuspended in 200 μL of 80% methanol before analysis.

2.2 LC-MS analysis

Serum metabolomic profiling was performed on a Vanquish UHPLC system (Thermo, Waltham, USA) coupled with a Q Exactive Orbitrap mass spectrometer (Thermo, Waltham, USA) operating in positive (ESI+) and negative (ESI-) modes. Samples were first eluted on an UPLC BEH Amide column (2.1 mm × 100 mm, 1.7

μm) at 25 ℃ with a flow rate of 0.3 mL/min. The mobile phase A was ultrapure water (pH: 9.75) containing 25 mM NH_4OH and 25 mM NH_4OAc and the mobile phase B was 25 mM acetonitrile. The gradient program was: 1) 0-0.5 min, 95% B; 2) 0.5-7.0 min, 95%-65% B; 3) 7.0-8.0 min, 65%-40% B, 4) 8.0-9.0 min, 40% B; 5) 9.0-9.1 min, 40%-95% B; 6) 9.1-12.0 min, 95% B. The auto-sampler temperature was 4 ˚C, and the injection volume was 3 μL.

To acquire MS/MS spectra, the column eluent was further detected by means of information-dependent acquisition on a Q Exactive Orbitrap Mass Spectrometer. The scan range was 60-1200 Da. The ion source parameters were as follows: sheath gas flow rate, 30 Arb; auxiliary gas flow rate, 25 Arb; capillary temperature, 350 ℃; full MS resolution, 60000; collision energy, 10, 30, or 60 V in ESI+ and -10, -30, or -60 V in ESI- modes, spray Voltage, 3.6 kV (ESI+) and -3.2 kV (ESI-).

3. Targeted metabolite analysis

3.1 Serum sample preparation

Briefly, 100 μL of serum samples was extracted in 400 μL of ice-cold methanol, centrifuged for 13,000 *g* at 4 ℃ for 10 min, and filtered through 3 kDa membrane cartridges. Sample extracts were then dried under vacuum, reconstituted in 200 μL of 100 mM NH_4OAc buffer, and capped before analysis.

3.2 LC-MS analysis

The targeted metabolite analysis of the 6 selected metabolites was conducted by a 20AD UPLC system (Shimadzu, Kyoto, Japan) coupled with a QTrap 5500 mass spectrometer (SCIEX, Framingham, USA), as described previously

[6]. Samples and standards (20 μL) were injected onto a Phenom-exex NH_2 column (150 mm × 2 mm × 3 μm) for metabolite separation. A binary solvent gradient consisting of 5 mM NH_4OAc (pH: 9.5) adjusted with ammonia (mobile phase A) and acetonitrile (mobile phase B) with a flow rate of 0.25 mL/min. Initial solvent composition at injection was 25% A, followed by a 2-min gradient to 45% A and a fast gradient ramp to 80% A (0.1 min) which was maintained for 5.9 min, A was increased again to 95% (2 min), held for 13 min and then reverted to initial conditions (0.1 min) for equilibration, with a total run time of 30 min.

The column flow was directed into the MS detector operated in the multiple reaction monitoring mode. The ion source parameters were as follows: sheath gas flow rate, 20 Arb; auxiliary gas flow rate, 10 Arb; capillary temperature, 300 ℃; capillary voltage, 4000 V. Other MS/MS settings, such as declustering potential and collision energy, were optimized for each particular metabolite. The concentrations of detected metabolites were obtained from 7-point calibration curves, which were constructed using the peak area ratios (peak area of the metabolite divided by peak area of the isotope-labeled internal standards) of each calibrator versus its concentration. All calibrators were purchased from Sigma (cat # 72340, cat # M4627, cat # C7344, cat # C0254, cat # P2263). All the involved internal standards, including NAM-D4 (for NAM, 1-MNAM, and ADPR, cat # DLM-6883-PK), L-tryptophan-D5 (cat # DLM-1092-PK), D-glucose-6-phosphate-C13 (for D-ribose-5-phosphate, cat # CLM-9601-PK), and 1,2-diheptanoyl-sn-glycero-3-phosphocholine-D26 (for PC(36:2), cat # DLM-11092-PK), were purchased from Cambridge Isotope

Laboratories. The calibration data of each detected metabolite, including calibration range, calibration linearity, regression equation, precision, and accuracy, were summarized in Table S1.

4. Machine learning algorithms

4.1 Random forest (RF)

The R package "randomForest" was used for this algorithm. A RF model of the 6 metabolic predictors was trained with 10-fold cross-validation. The 2 important RF parameters, nTree (number of trees to grow for each forest) and mTry (number of predictors sampled for splitting at each node), were set to 500 and the default setting, respectively.

4.2 Extreme Gradient Boosting (XGBoost) tree

The R package "xgboost" was used for this algorithm. An XGBoost tree model of the 6 metabolic predictors was trained with 10-fold cross-validation. Key parameters were set as follows: eta, 0.05; max_depth, 20; min_child_weight, 1 (default); nrounds, 100; objective, "binary: logistic"; eval_metric, "auc".

4.3 Support Vector Machines (SVM)

The SVM model was fitted with the R package "e1071". The kernel parameter was set as "polynomial", which showed the best predictive performance on the discovery set among all kernel types. We set the "cost" parameter as 0.3, the "gamma" parameter as 1, the "degree" parameter as 4, and the "coef0" parameter as 1.

4.4 Deep neural network (DNN)

We trained a deep, multilayer neural network with 2 hidden layers using the R package "keras". We used a rectified linear unit function as the non-linear activation function and a sigmoid function as the classification function. Model optimization was guided using the adaptive moment estimation optimizer. The hyperparameters were manually tuned as follows: regularization factor, 0.002; learning decay rate for the first momentum, 0.9; for the second momentum, 0.999; batch size, 1024; epochs, 30. The DNN was designed as having 2 hidden layers with 32 input units in the first layer and 16 input units in the second layer. Ten percent of the input units were used as the drop-out layers, which evaluate the cross-entropy loss function over the number of epochs and reduce the learning rate when the loss function stopped improving.

5. In vitro experiments

5.1 NAD^+ detection by LC-MS

Prior to NAD^+ detection, high glucose-cultured HASMCs (LONZA, cat # CC-2571) were trypsinised, washed, and seeded to 24-well plates at 10^5 cells per well. The processes of sample preparation, LC separation, and MS detection were the same as the target metabolite analysis mentioned above. The isotope-labeled internal standard used for NAD^+ detection was adenosine monophosphate C13 (Cambridge Isotope Laboratories, cat # CNLM-3802-SL-10), as described by Grant et al [6].

5.2 Determination of mitochondrial complex activity

HASMCs were fractionated by mitochondria isolation kit (Sigma, cat # MITOISO2-1KT). The activity of mitochondrial respiratory chain complexes (I-V) in mitochondria fractions was determined by spectrophotometric methods summarized

by Rodenburg RJ [7].

5.3 Bioenergetic profiles detected by the Seahorse technology

The Seahorse XFe96 Analyzer (Agilent, Santa Clara, USA) was used to simultaneously detect mitochondrial respiration and glycolysis of HAoSMCs by generating the time course of oxygen consumption rate (OCR) and extracellular acidification rate (ECAR), respectively. At first, HASMCs (6×10^5 cells per well) were plated into a Seahorse XF24 Cell Culture Microplate (Agilent) filled with high glucose DMEM, and treated with 10 μM FK866 (Sigma, cat # F8557), 200 μM 1MT (Sigma, cat # 447439), or 10 μM NMN (Sigma, cat # N3501) for 20 h.

For measurement of mitochondrial respiration, the medium was replaced with DMEM containing 1 mM pyruvate, 2 mM glutamine, and 25 mM glucose prior to assays. Then, cells were sequentially exposed to 1 μM oligomycin (Oligo, Abcam Biochemicals, cat # 1404-19-9), 0.75 μM FCCP (Sigma, cat # C2920), and a mix of 1 μM each rotenone (Sigma, cat # R8875) and antimycin (Sigma, cat # A8674) (R + A) at the indicated times, and the OCRs of HASMCs were measured over time (as presented in Fig. 5C). According to the manufacturer's protocol, non-mitochondrial OCR was calculated as OCR after R + A injection; Basal respiration was calculated as the difference between the OCR before Oligo injection and non-mitochondrial OCR; ATP-linked respiration was calculated as the difference between basal and Oligo-inhibited OCR. Maximal respiration was calculated as the difference between FCCP-induced OCR and non-mitochondrial respiration. Reserve respiratory capacity was calculated as the difference between maximal and basal respiration.

For measurement of glycolytic flux, the medium was replaced with glucose-free DMEM prior to assays. Then, cells were sequentially exposed to 19 mM glucose, 1 μM Oligo, and 50 mM 2-deoxy-glucose (Sigma, cat # D6134) at the indicated times (as presented in Fig. 5E), and the ECARs of cells were measured over time. According to the manufacturer's protocol, non-glycolytic acidification was calculated as ECAR before glucose injection; glycolysis was calculated as the difference between glucose-induced ECAR and non-glycolytic acidification; glycolytic capacity was calculated as the difference between Oligo-induced ECAR and non-glycolytic acidification; glycolytic reserve was calculated as the difference between glycolytic capacity and glycolysis. All the readings of OCRs and ECARs were normalized to the total protein content of each well (determined by the Bradford method [8]).

5.4 Reverse-transcription quantitative PCR (RT-qPCR)

Total RNA was extracted from HASMCs using the Trizol reagent (Sigma, cat # 93289). cDNA was synthesized with RNA to cDNA EcoDry Premix (Clontech, cat # 639547). The mRNA expression of proinflammatory genes was determined by RT-qPCR on a CFX96 Touch system (Bio-rad, Hercules, USA). RT-qPCR was performed in triplicate based on the MIQE guidelines [9]. The relative expression of a target gene was normalized to the expression of reference gene (*GAPDH*) using the $2^{-\triangle\triangle Cq}$ method [10]. The calibrator was a cDNA sample from the control group. The primers used for RT-qPCR analysis are listed in Table S2.

5.5 Transwell migration assay

HASMCs were seeded into 24-well plates, with administration of FK866, 1MT,

or NMN as mentioned above. Transwell inserts with 8 μm pores (Sigma, cat # CLS9668) were pre-coated with 10 μg/mL fibronectin for 24 h, and applied in suspension. THP1 monocytes were labeled with Qdot (Invitrogen, cat # Q10141MP) following the manufacturer's instructions, and then added to the top of each transwell. After 4 h incubation, transwell inserts were taken, and un-migrated monocytes on the upper membrane were removed by gentle rubbing with a cotton bud. Migrated Qdot-labeled THP1 cells were enumerated by fluorescence microscopy.

5.6 Proliferation assay

HASMC proliferation was assessed by a methylene blue assay [11]. Briefly, HASMCs (1.5×10^5 cells/well) were placed in sterile 96-well culture plates and cultured in DMEM, with administration of FK866 (10 μM), 1MT (200 μM), or NMN (10 μM) for 20 h. Cells were then fixed in 10% formaldehyde saline for 30 min, and incubated with 1 % (*w/v*) methylene blue in 0.01 mM borate buffer (pH 8.5) for 30 min. The remaining dye was washed off by serially dipping the plate into each of four tanks of 0.01 mol/L borate buffer (pH 8.5). The resulting cell growth was measured at 540 nm and calculated in comparison with the growth of the control group.

Supplementary Figures

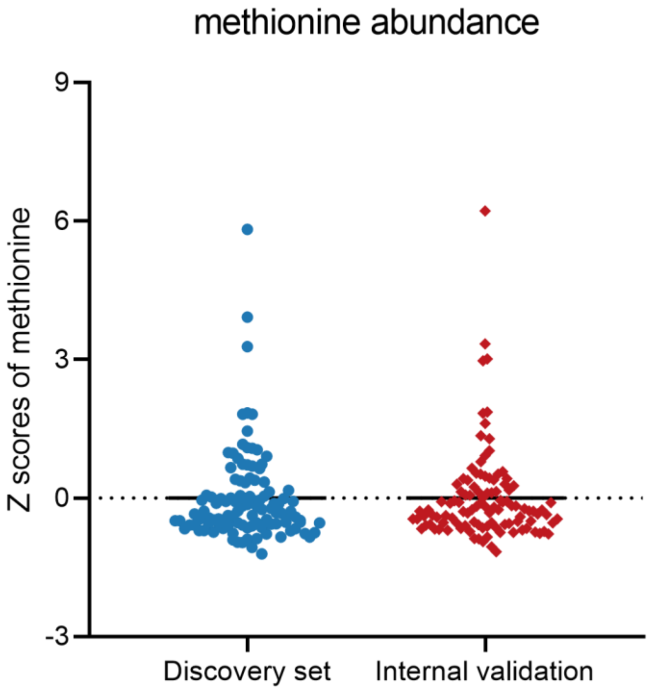

Figure S1 The Z distribution of methionine abundance in both discovery and internal validation sets. The Z scores of methionine across all samples were greater than -3 (-3SD), indicating that the serum samples were properly stored for metabolite detection.

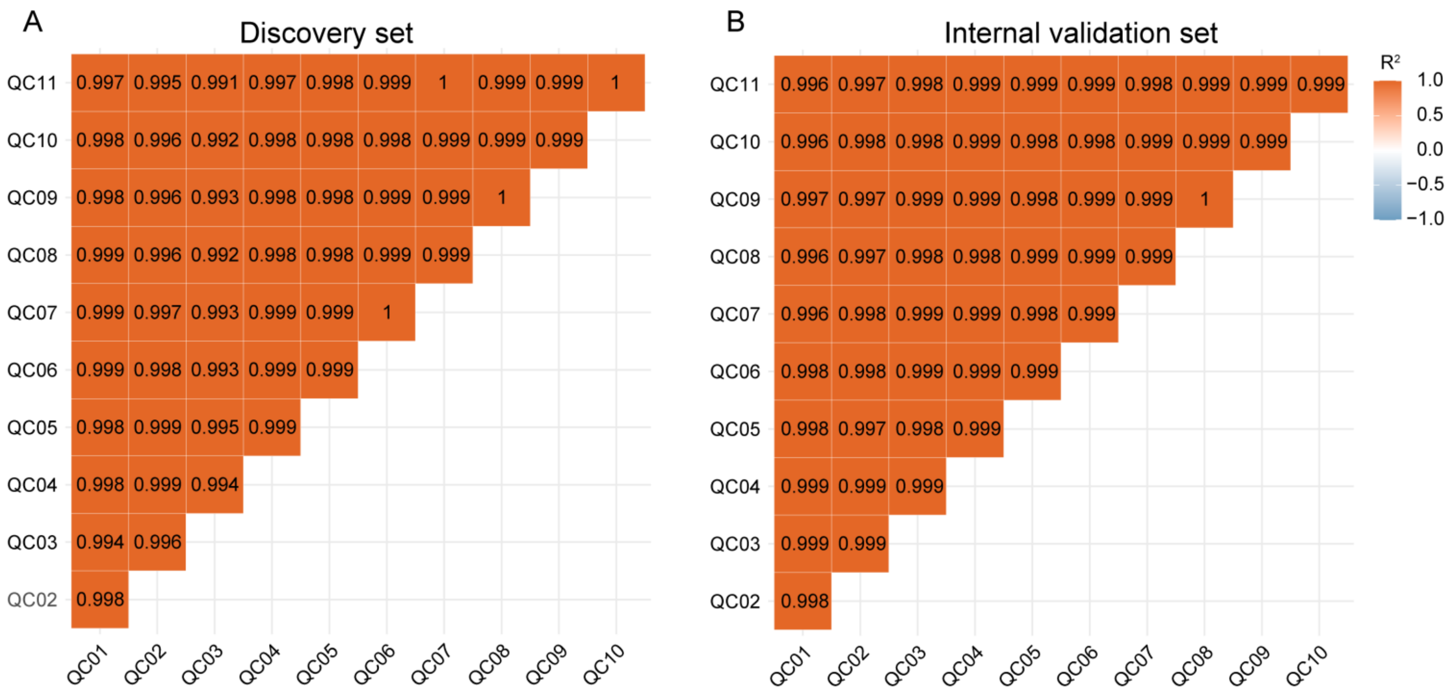

Figure S2 The Pearson correlation between metabolic data of quality control samples assessing the reliability of the LC-MS analysis in the discovery (**A**) and internal validation sets (**B**).

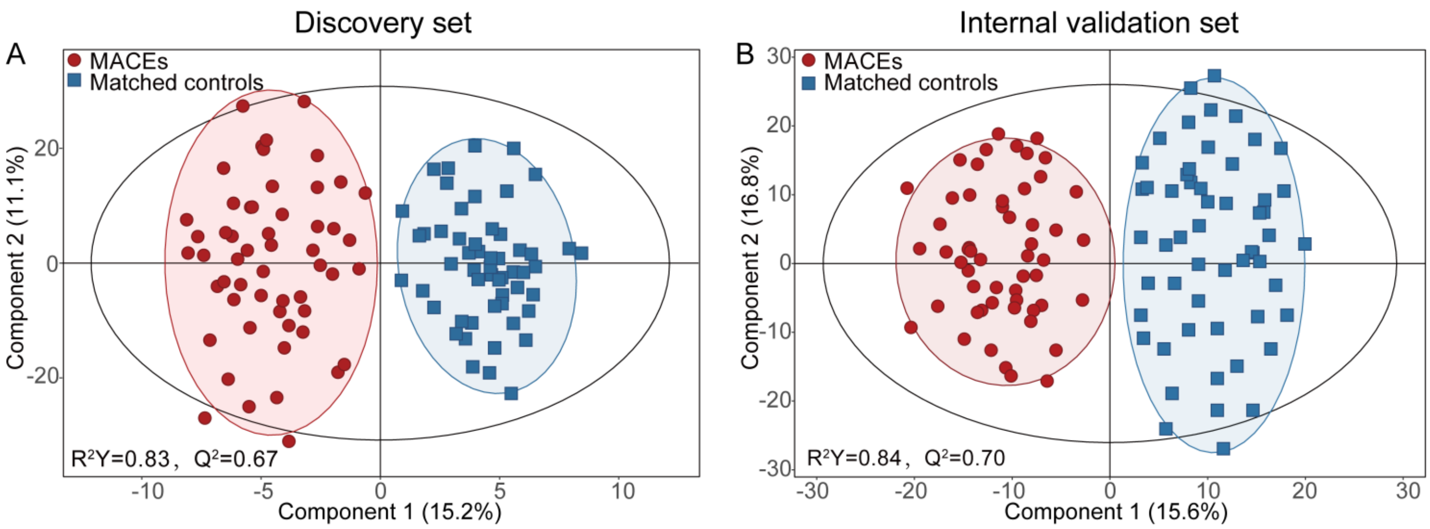

Figure S3 The OPLS-DA analysis assessing the performance of 35 differential metabolites for discrimination of MACEs from matched controls in the discovery (**A**) and internal validation sets (**B**).

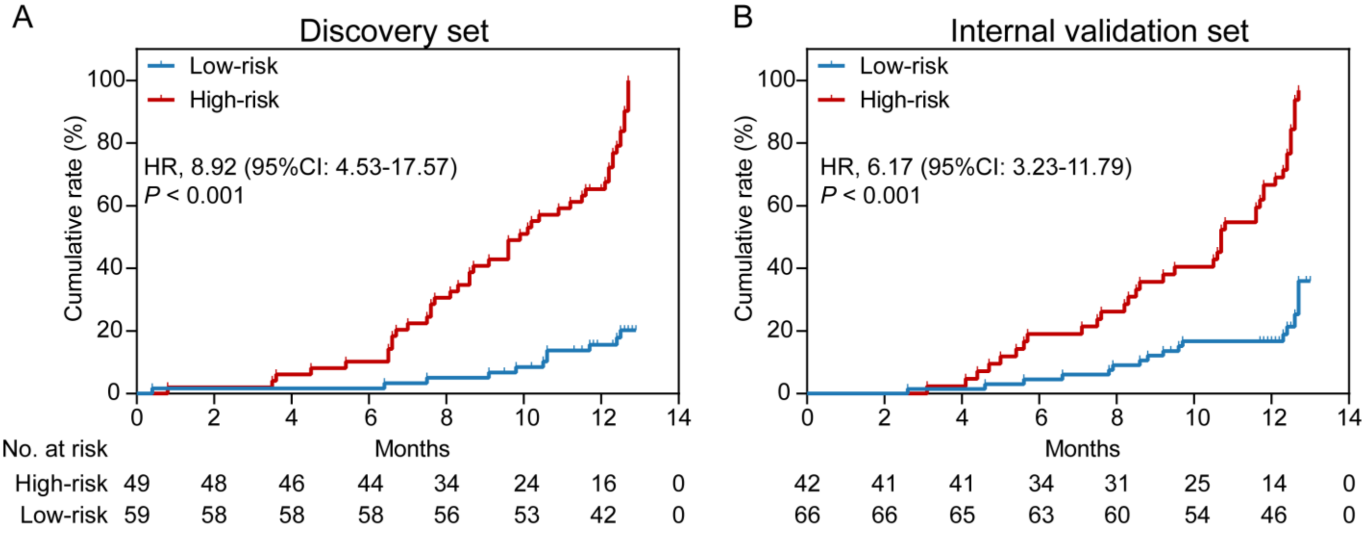

Figure S4 The differences in cumulative rates of MACEs between participants with high-risk and low risk scores of the 6-metabolite signatures. *P* values were derived from Cox regression with adjustment for age, sex, smoking status, obesity (BMI > 25 kg/m^2), hypertension, HbA1c, LVEF < 50%, clinical presentations, multivessel CAD, SYNTAX score, and stent types.

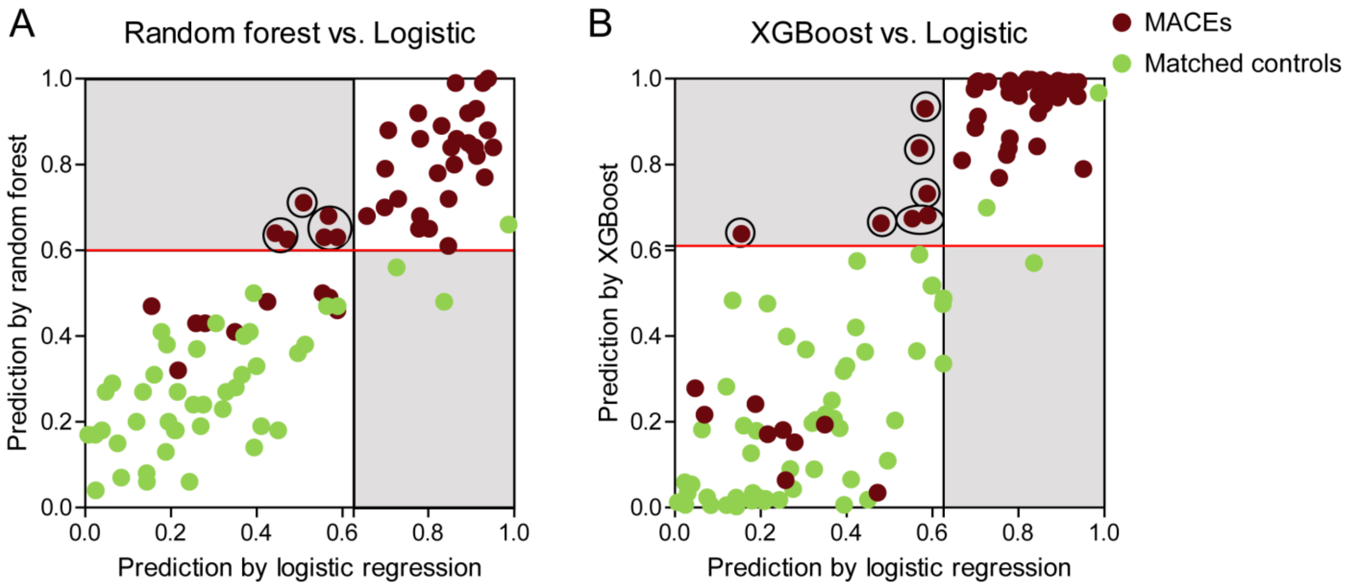

Figure S5 Scatter plots for comparing the predictive performance of the random forest (**A**) and XGBoost (**B**) models to the logistic regression model of the 6-metabolite panel. The models are generated using the discovery dataset and presented here in the internal validation set. Red lines indicate the cut-offs of random forest and XGBoost models; Black lines indicate the cut-off of logistic regression model. Black circles label MACEs that would be identified using the random forest and XGBoost models but would be missed when the logistic regression model is applied

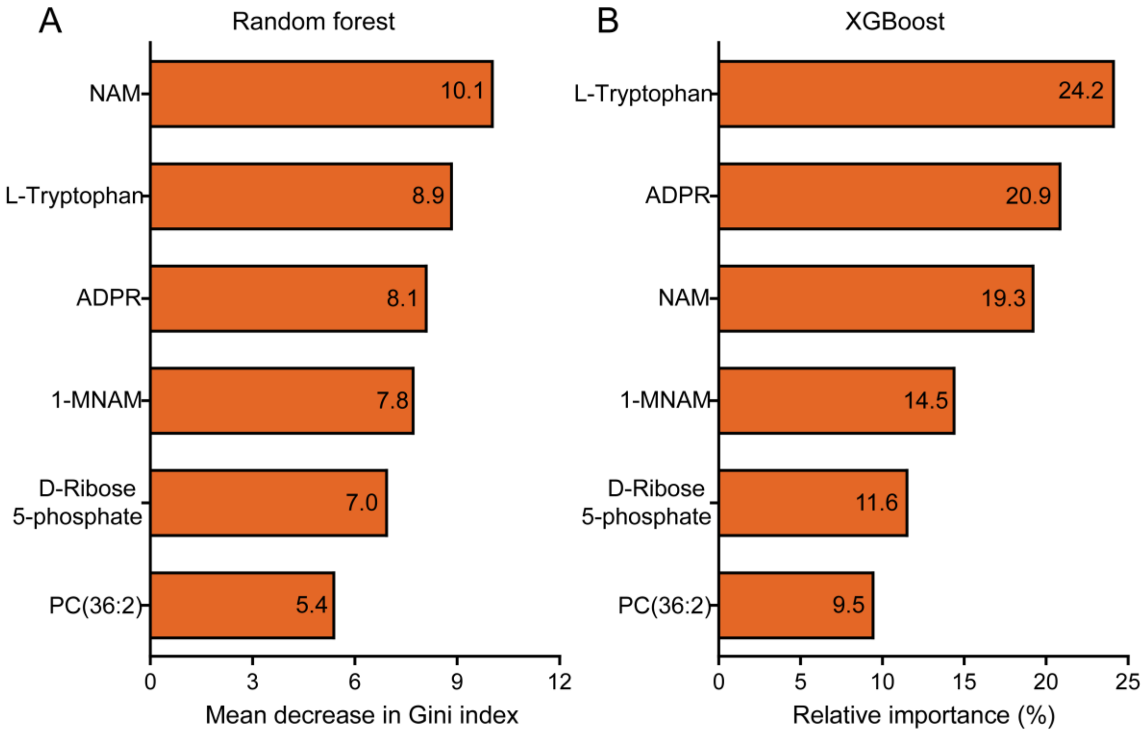

Figure S6 The importance of each predictor of the 6-metabolite classifier constructed by random forest (**A**) and XGBoost (**B**). Abbreviations: NAM, nicotinamide; ADPR, adenosine diphosphate ribose; 1-MNAM, 1-methylnicotinamide; PC, phosphatidylcholine.

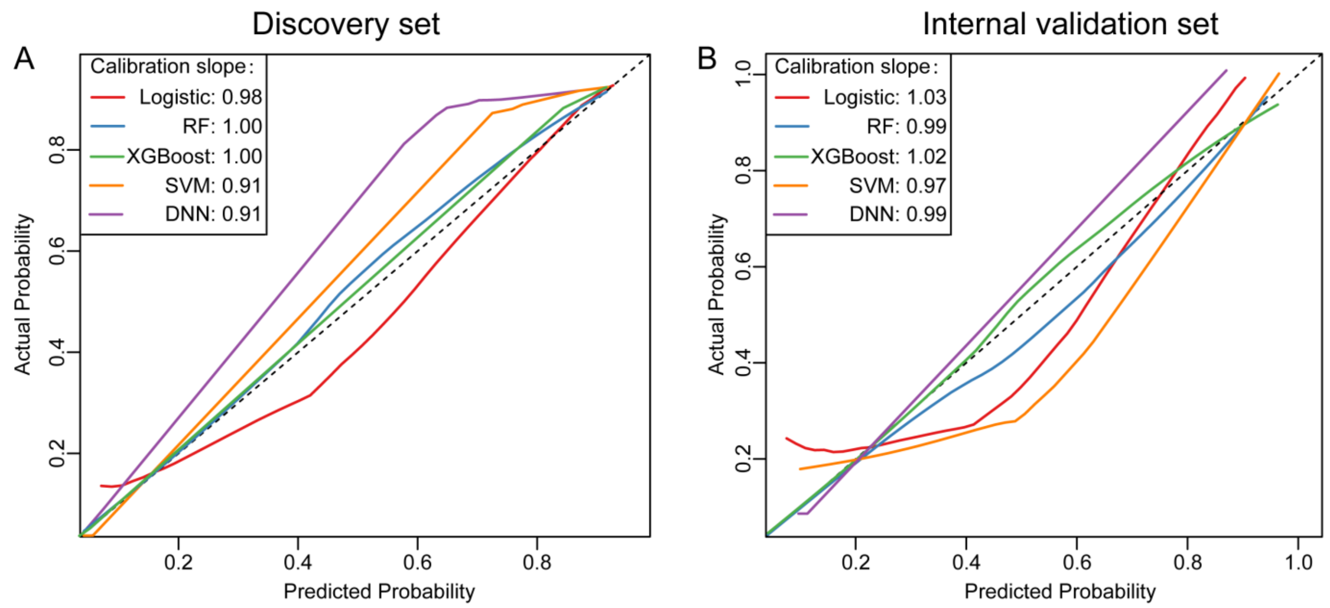

Figure S7 Calibration plots for the logistic regression and 4 machine learning models of the 6-metabolite classifier in the discovery (**A**) and internal validation sets (**B**). Abbreviations: RF, random forest; XGBoost, extreme gradient boosting; SVM, Support Vector Machines; DNN, deep neural network.

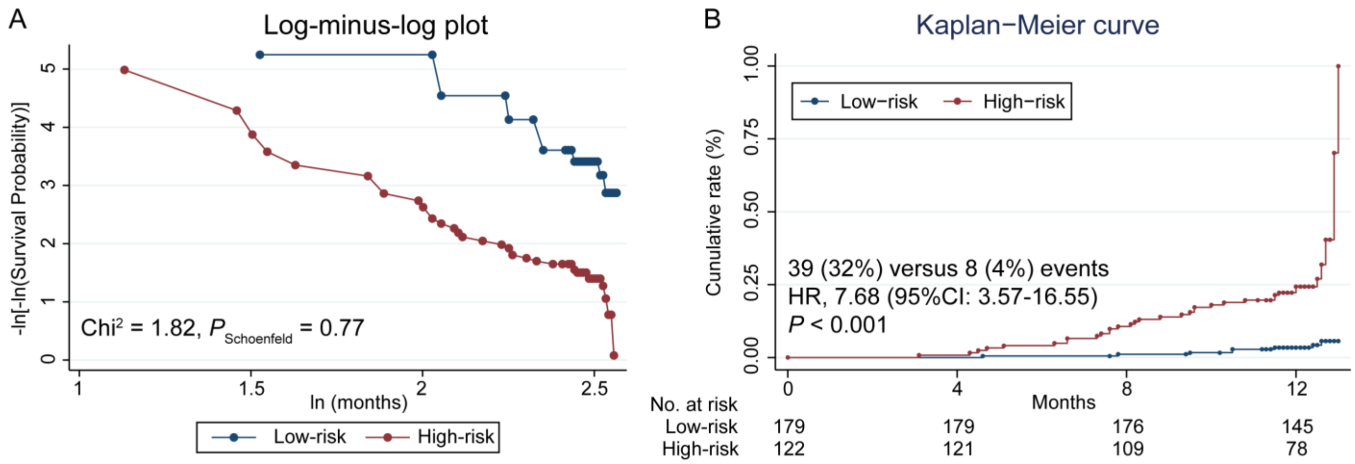

Figure S8 Backward stepwise Cox regression analyses of the association between the 6-metabolite classifier and MACEs in the external validation set. (A) The log-minus-log plot for graphically testing the proportional hazards assumption. (B) Kaplan-Meier curve for assessing the performance of the 6-metabolite classifier to predict MACEs. In the backward stepwise Cox regression analyses, variables including age, sex, smoking status, obesity (BMI > 25 kg/m^2), hypertension, HbA1c, LVEF < 50%, clinical presentations, multivessel CAD, SYNTAX score, stent types, and the 6-metabolite classifier were first entered one at a time. Then, 4 variables with *P*< 0.10 (i.e. HbA1c, LVEF < 50%, multivessel CAD, and the 6-metabolite classifier) in the stepwise procedure were retained to fit the final model. The HR and P value were calculated accordingly.

Supplementary Tables

Table S1

Calibration data for 6 metabolites detected by targeted metabolite analyses.

Metabolites	Calibration range (μmol/L)	Regression equation	Linearity (r^2)	Intra-batch precision (%)	Inter-batch precision (%)	Accuracy (%)
ADPR	0-0.8	$y = 5.0\times10^{-3}x+7.6\times10^{-1}$	0.9979	9.6	5.4	4.1
1-MNAM	0-0.8	$y = 8.4\times10^{-3}x+7.0\times10^{-1}$	0.9938	13.7	8.8	-4.2
D-Ribose 5-phosphate	0-2.1	$y = 4.7\times10^{-3}x+1.6\times10^{-2}$	0.9958	5.8	3.4	-9.1
NAM	0-0.8	$y = 7.5\times10^{-3}x+8.2\times10^{-3}$	0.9937	6.0	3.8	3.4
L-Tryptophan	10-510	$y = 8.3\times10^{-5}x+5.4\times10^{-5}$	0.9942	13.1	7.6	-2.0
PC(36:2)	0-640	$y = 6.8\times10^{-3}x+1.8\times10^{-1}$	0.9961	10.4	6.4	5.3

Abbreviation: ADPR, adenosine diphosphate ribose; 1-MNAM, 1-methylnicotinamide; NAM, nicotinamide; PC, phosphatidylcholine

Table S2

List of primers used in RT-qPCR.

Gene	Primers	Length (bp)	Ann. Temp (℃)
MCP1	Forward: GCTCAGCCAGATGCAATCA	193	56
	Reverse: TGGAATCCTGAACCCACTT		
CCL3	Forward: CCCTTGCTGTCCTCCTCTG	243	58
	Reverse: GGTCGCTGACATATTTCTGG		
CCL4	Forward: AGCCCGGATGCTTCTCCA	113	58
	Reverse: CCTAATACAATAACACGGCACATAA		
IL6	Forward: GCCTGCGTCCGTAGTTTCC	179	60
	Reverse: CTCTTTCGTTCCCGGTGGG		
IL8	Forward: TTGGCAGCCTTCCTGATTT	217	58
	Reverse: CCTTGGGGTCCAGACAGAG		
IL1β	Forward: GATGGCTTATTACAGTGGCA	139	56
	Reverse: GTAGTGGTGGTCGGAGATT		
CCL7	Forward: TTCAACTACCTGCTGCTAC	171	54
	Reverse: ATAAAGTCCTGGACCCACT		
CXCL2	Forward: AACCGAAGTCATAGCCACA	149	56
	Reverse: AGGAACAGCCACCAATAAG		
CX3CL1	Forward: TGTAGCTTTGCTCATCCACTATC	246	58
	Reverse: AACCACAGACTCGTCCATTCC		
IL1α	Forward: TGTATGTGACTGCCCAAGA	258	56
	Reverse: TGAGACTCCAGACCTACGC		
TNFα	Forward: CCCATGTTGTAGCAAACCC	223	58
	Reverse: TGGTAGGAGACGGCGATGC		
PTGS2	Forward: CTTCCTCCTGTGCCTGATG	172	58
	Reverse: ACTGATGCGTGAAGTGCTG		
GAPDH	Forward: GAAGGTGAAGGTCGGAGTC	226	58
(Reference)	Reverse: GAAGATGGTGATGGGATTTC		

Table S3

Ontology classes of metabolites in the discovery and internal validation sets.

Ontology class	Discovery set (n = 743)	Internal validation set (n = 674)
Lipids, fatty acids and related	320	305
Energy metabolism and related	100	93
Carbohydrates and related	94	86
Amino acids and related	88	74
Peptides, and analogues	37	29
Signal substances and related	34	30
Vitamins and related	27	21
Nucleosides and related	26	20
Alkaloids and derivatives	9	9
Miscellaneous	8	7

Table S4

69 differential metabolites in the discovery set.

ID	Metabolite name	Subpathway	Superpathway	VIP	*P* -VALUE	FDR	Fold Change
**1**	**Indolepyruvate**	**Tryptophan metabolism**	**Amino acid**	**2.22**	**1.09E-03**	**5.99E-03**	**5.36**
**2**	**Acetate**	**Glycolysis / Gluconeogenesis**	**Carbohydrates and related**	**2.04**	**2.07E-03**	**9.77E-03**	**4.62**
**3**	**ADPR**	**Nicotinate and nicotinamide metabolism**	**Energy metabolism**	**2.64**	**5.49E-10**	**1.06E-07**	**4.61**
4	beta-D-Glucoside	Starch and sucrose metabolism	Carbohydrates and related	2.51	7.13E-08	5.04E-06	3.88
5	Choline sulfate	ABC transporters	Signal substances and related	1.68	3.20E-03	1.35E-02	3.63
**6**	**Tryptamine**	**Tryptophan metabolism**	**Amino acid**	**1.24**	**1.11E-03**	**6.07E-03**	**2.59**
7	L-Serine-phosphoethanolamine	Glycerophospholipid	Lipids	2.20	7.49E-07	2.13E-05	2.29
**8**	**(S)-Lactate**	**Glycolysis**	**Carbohydrates and related**	**1.62**	**2.86E-06**	**5.93E-05**	**2.22**
9	3-Carboxy-4-methyl-5-propyl-2-furanpropionic acid	Fatty acid metabolism	Lipids	1.51	9.47E-03	2.98E-02	2.19
10	2,6-Dihydroxypyridine	Nicotine degradation	Energy metabolism	2.16	1.68E-04	1.45E-03	1.99
11	Desglucocheirotoxin	Steroid lactones	Lipids	2.09	4.32E-04	2.96E-03	1.94
12	5-Amino pentanamide	Lysine degradation	Amino acid	2.12	5.58E-04	3.57E-03	1.86
13	LysoPC(C18:1)	Glycerophosphocholines	Lipids	1.41	5.57E-05	6.08E-04	1.86
**14**	**5-Carboxamide**	**Nicotinate and nicotinamide metabolism**	**Energy metabolism**	**2.22**	**1.55E-03**	**7.83E-03**	**1.85**
**15**	**1-MNAM**	**Nicotinate and nicotinamide metabolism**	**Energy metabolism**	**2.56**	**5.53E-09**	**7.79E-07**	**1.80**
16	Phenylacetylglutamine	Phenylalanine metabolism	Amino acid	1.06	9.15E-03	2.90E-02	1.79
17	Indole-3-acetamide	Tryptophan metabolism	Amino acid	2.02	1.75E-06	4.04E-05	1.71
18	Oleic acid	Fatty acid metabolism	Lipids	1.98	6.16E-04	3.84E-03	1.63
19	dUMP	Pyrimidine deoxyribonucleotides	Nucleosides and related	1.84	9.83E-05	9.49E-04	1.62
**20**	**PA(34:1)**	**Phosphatidic acid**	**Lipids**	**2.09**	**1.10E-05**	**1.65E-04**	**1.59**
**21**	**Indole-3-acetate**	**Tryptophan metabolism**	**Amino acid**	**1.83**	**8.23E-05**	**8.29E-04**	**1.57**
**22**	**D-Ribose 5-phosphate**	**Pentose phosphate**	**Carbohydrates and related**	**2.73**	**1.27E-10**	**3.25E-08**	**1.57**
**23**	**Pyruvic acid**	**Glycolysis / Gluconeogenesis**	**Carbohydrates and related**	**1.64**	**7.44E-05**	**7.66E-04**	**1.57**
24	Arachidonic acid	Fatty acid metabolism	Lipids	1.12	1.46E-02	4.06E-02	1.57
**25**	**PC(33:3)**	**Phosphatidylcholine**	**Lipids**	**1.50**	**1.09E-03**	**5.97E-03**	**1.54**
**26**	**Cysteine-S-sulfate**	**Cysteine and methionine metabolism**	**Amino acid**	**1.88**	**2.25E-04**	**1.81E-03**	**1.50**
27	2-Methylguanosine	Purine nucleosides	Nucleosides and related	1.82	3.76E-03	1.52E-02	1.45
**28**	**D-ribose**	**Pentose phosphate**	**Carbohydrates and related**	**1.03**	**9.89E-03**	**3.07E-02**	**1.44**
**29**	**PC(34:2)**	**Phosphatidylcholine**	**Lipids**	**2.02**	**2.25E-05**	**2.96E-04**	**1.43**
**30**	**3-Phospho-D-glycerate**	**Pentose phosphate**	**Carbohydrates and related**	**1.88**	**4.00E-03**	**1.59E-02**	**1.42**
31	Lactosylceramide (d18:1/16:0)	Glycosphingolipids	Lipids	1.68	1.32E-03	6.94E-03	1.40

**32**	**Propionic acid**	**Nicotinate and nicotinamide metabolism**	**Energy metabolism**	**1.93**	**7.58E-05**	**7.77E-04**	**1.40**
**33**	**8-Methoxykynurenate**	**Tryptophan metabolism**	**Amino acid**	**1.05**	**1.38E-02**	**3.89E-02**	**1.40**
**34**	**PC(33:4)**	**Phosphatidylcholine**	**Lipids**	**2.47**	**1.11E-06**	**2.77E-05**	**1.38**
35	Dihydrolipoate	Fatty acid metabolism	Lipids	2.14	1.17E-03	6.31E-03	1.35
**36**	**L-Acetylcarnitine**	**Fatty acid metabolism**	**Lipids**	**1.36**	**4.19E-03**	**1.64E-02**	**1.34**
**37**	Hexadecylglycero-3-phosphocholine	Glycerophosphocholines	Lipids	2.40	3.98E-07	1.42E-05	1.34
**38**	**Nicotinamide riboside**	**Nicotinate and nicotinamide metabolism**	**Energy metabolism**	**2.18**	**1.85E-05**	**2.51E-04**	**0.74**
39	7-Dehydrodesmosterol	Cholestane steroids	Lipids	1.50	1.13E-05	1.68E-04	0.74
40	PC(38:3)	Phosphatidylcholine	Lipids	1.94	1.23E-04	1.13E-03	0.74
41	3-Methylhistidine	Histidine metabolism	Amino acid	1.86	1.59E-04	1.39E-03	0.73
**42**	**Nicotinate**	**Nicotinate and nicotinamide metabolism**	**Energy metabolism**	**1.93**	**7.64E-04**	**4.54E-03**	**0.73**
43	PE(40:4)	Phosphatidylethanolamine	Lipids	1.86	5.05E-04	3.31E-03	0.73
44	SM(d18:0/14:0)	Phosphosphingolipids	Lipids	1.65	1.92E-03	9.21E-03	0.73
45	Androsterone sulfate	Sulfated steroids	Lipids	1.05	1.33E-02	3.81E-02	0.72
**46**	**PC(36:2)**	**Phosphatidylcholine**	**Lipids**	**2.64**	**4.01E-07**	**1.42E-05**	**0.71**
47	PE(36:2)	Phosphatidylethanolamine	Lipids	1.91	6.53E-05	6.89E-04	0.70
**48**	**SM C18:1**	**Phosphosphingolipids**	**Lipids**	**1.97**	**2.54E-04**	**1.99E-03**	**0.69**
49	LysoPC(C22:0)	Glycerophosphocholines	Lipids	2.34	3.45E-08	3.07E-06	0.69
50	L-Hexanoylcarnitine	Fatty acid esters	Lipids	1.70	1.96E-02	4.96E-02	0.69
51	LysoPC(C14:0)	Glycerophosphocholines	Lipids	1.19	7.82E-04	4.62E-03	0.68
**52**	**Trigonelline**	**Nicotinate and nicotinamide metabolism**	**Energy metabolism**	**2.28**	**3.96E-07**	**1.41E-05**	**0.67**
**53**	**Quinolinate**	**Tryptophan metabolism**	**Amino acid**	**1.52**	**8.70E-03**	**2.79E-02**	**0.67**
**54**	**6-Hydroxynicotinate**	**Nicotinate and nicotinamide metabolism**	**Energy metabolism**	**1.68**	**5.78E-05**	**6.26E-04**	**0.66**
55	PC(34:4)	Phosphatidylcholine	Lipids	1.40	7.58E-03	2.53E-02	0.65
**56**	**L-Tryptophan**	**Tryptophan metabolism**	**Amino acid**	**3.04**	**1.02E-12**	**7.78E-10**	**0.64**
**57**	**3-Indoleacetonitrile**	**Tryptophan metabolism**	**Amino acid**	**1.72**	**5.38E-04**	**3.47E-03**	**0.64**
**58**	**Xanthine**	**Purine metabolism**	**Nucleosides and related**	**2.57**	**1.73E-07**	**9.05E-06**	**0.64**
**59**	**NAM**	**Nicotinate and nicotinamide metabolism**	**Energy metabolism**	**2.66**	**1.12E-08**	**1.25E-06**	**0.60**
60	Dodecanoic acid	Fatty acid metabolism	Lipids	1.43	1.65E-04	1.43E-03	0.60
61	UDP-D-xylose	Nucleotide sugar metabolism	Carbohydrates and related	2.14	2.62E-05	3.34E-04	0.58
**62**	**3-Hydroxy-L-kynurenine**	**Tryptophan metabolism**	**Amino acid**	**2.08**	**1.09E-04**	**1.03E-03**	**0.58**
63	2-Aminomuconic acid	Tryptophan metabolism	Amino acid	1.34	6.98E-03	2.38E-02	0.56
64	PC(28:0)	Phosphatidylcholine	Lipids	1.73	3.16E-03	1.34E-02	0.56
65	Sphinganine	Sphingolipid metabolism	Lipids	2.71	1.16E-07	6.99E-06	0.52
**66**	**PE(38:6)**	**Phosphatidylethanolamine**	**Lipids**	**2.00**	**8.72E-04**	**5.03E-03**	**0.50**
67	Apigenin 8-C-glucoside	Flavonoid biosynthesis	Lipids	2.62	3.18E-05	3.89E-04	0.49
**68**	**PC(38:6)**	**Phosphatidylcholine**	**Lipids**	**2.70**	**5.34E-07**	**1.73E-05**	**0.39**
**69**	**L-Kynurenine**	**Tryptophan metabolism**	**Amino acid**	**2.61**	**6.35E-06**	**1.11E-04**	**0.33**

Bold values indicate metaboites differentially expressed in both discovery and internal validation sets.

Table S5
89 differential metabolites in the internal validation set.

ID	Metabolite name	Subpathway	Superpathway	VIP	*P* -VALUE	FDR	Fold Change
**1**	**Indolepyruvate**	**Tryptophan metabolism**	**Amino acid**	**1.95**	**2.13E-03**	**9.05E-03**	**3.68**
**2**	**PC(33:4)**	**Phosphatidylcholine**	**Lipids**	**2.12**	**1.47E-05**	**2.41E-04**	**3.62**
**3**	**Acetate**	**Glycolysis / Gluconeogenesis**	**Carbohydrates and related**	**1.72**	**3.46E-03**	**1.30E-02**	**3.60**
**4**	**ADPR**	**Nicotinate and nicotinamide metabolism**	**Energy metabolism**	**2.48**	**5.88E-04**	**3.53E-03**	**3.07**
**5**	**Indole-3-acetate**	**Tryptophan metabolism**	**Amino acid**	**1.48**	**2.81E-04**	**2.03E-03**	**2.69**
**6**	**PC(34:2)**	**Phosphatidylcholine**	**Lipids**	**2.27**	**2.86E-04**	**2.05E-03**	**2.69**
**7**	**(S)-Lactate**	**Glycolysis**	**Carbohydrates and related**	**1.69**	**7.77E-08**	**9.67E-06**	**2.62**
**8**	**Tryptamine**	**Tryptophan metabolism**	**Amino acid**	**1.26**	**3.50E-03**	**1.31E-02**	**2.52**
9	Deoxycholic acid	Bile acids and derivatives	Lipids	1.14	1.65E-04	1.39E-03	2.47
10	Phenylalanyl-Methionine	Methionine metabolism	Amino acid	1.18	6.34E-04	3.74E-03	2.40
11	Glycyl-Valine	Valine metabolism	Amino acid	1.77	1.56E-04	1.33E-03	2.26
12	N-Acetylhistidine	Histidine metabolism	Amino acid	1.32	1.96E-02	4.51E-02	2.14
13	Succinic acid semialdehyde	Fatty acid metabolism	Lipids	1.08	3.07E-04	2.16E-03	2.14
**14**	**D-ribose**	**Pentose phosphate**	**Carbohydrates and related**	**1.76**	**2.88E-04**	**2.06E-03**	**2.00**
15	5-Hydroxymethyldeoxycytidylate	Pyrimidine metabolism	Nucleosides and analogues	1.66	1.54E-06	4.92E-05	1.95
16	Carnosine	Histidine metabolism	Amino acids and analogues	1.94	5.18E-07	2.40E-05	1.94
17	Vitamin A	Retinol metabolism	Vitamins	1.89	1.09E-05	1.91E-04	1.90
18	Asparaginyl-Valine	Valine metabolism	Amino acid	1.77	1.27E-04	1.14E-03	1.82
**19**	**1-MNAM**	**Nicotinate and nicotinamide metabolism**	**Energy metabolism**	**2.58**	**5.65E-07**	**2.55E-05**	**1.79**
20	1-Methylinosine	Purine metabolism	Nucleosides and analogues	1.71	3.57E-03	1.33E-02	1.79
21	Formiminoglutamic acid	Histidine metabolism	Amino acid	1.44	4.86E-03	1.65E-02	1.71
22	bicyclo-PGE2	Eicosanoids	Lipids	1.16	8.39E-03	2.46E-02	1.70
23	Deoxyinosine	Purine metabolism	Nucleosides and related	1.53	4.43E-03	1.54E-02	1.67
24	L-lysine	Lysine metabolism	Amino acid	1.96	3.97E-08	7.35E-06	1.66
25	Aminoadipic acid	Lysine biosynthesis	Organic acids and derivatives	1.31	1.82E-02	4.29E-02	1.65
26	D-Glucuronic acid	Pentose and glucuronate interconversions	Carbohydrates and related	1.92	6.51E-03	2.06E-02	1.64
**27**	**8-Methoxykynurenate**	**8-Methoxykynurenate**	**Lipids and lipid-like molecules**	**2.17**	**1.83E-05**	**2.83E-04**	**1.64**
**28**	**Pyruvic acid**	**Glycolysis / Gluconeogenesis**	**Carbohydrates and related**	**1.79**	**2.22E-04**	**1.72E-03**	**1.63**
29	LysoPC(C18:3)	Glycerophosphocholines	Lipids	1.75	1.24E-03	5.99E-03	1.63
30	Hippuric acid	Phenylalanine metabolism	Amino acids and analogues	1.22	1.50E-02	3.77E-02	1.60
31	Phenylalanine	Phenylalanine metabolism	Amino acids and analogues	1.66	2.37E-04	1.80E-03	1.58
32	Phenylalanyl-Isoleucine	Isoleucine metabolism	Amino acid	1.80	3.67E-05	4.57E-04	1.57
33	PE(38:4)	Phosphatidylethanolamine	Lipids	2.23	2.64E-04	1.94E-03	1.57
34	Trimethylamine N-oxide	Methane metabolism	Organic nitrogen	1.23	5.09E-03	1.71E-02	1.55

**35**	**Propionic acid**	**Nicotinate and nicotinamide metabolism**	**Energy metabolism**	**1.54**	**3.74E-05**	**4.63E-04**	**1.54**
**36**	**D-Ribose 5-phosphate**	**Pentose phosphate**	**Carbohydrates and related**	**1.91**	**2.59E-03**	**1.05E-02**	**1.50**
37	L-Serine	Serine metabolism	Amino acid	2.18	1.71E-06	5.23E-05	1.50
38	Citrulline	Arginine biosynthesis	Amino acid	1.62	3.42E-05	4.35E-04	1.48
39	L-Asparagine	Asparagine metabolism	Amino acid	2.22	4.19E-07	2.05E-05	1.48
40	5'-Methylthioadenosine	Cysteine and methionine metabolism	Amino acids and analogues	1.67	1.25E-03	6.06E-03	1.47
**41**	**5-carboxamide**	**Nicotinate and nicotinamide metabolism**	**Energy metabolism**	**1.97**	**1.34E-02**	**3.48E-02**	**1.46**
42	PC(42:2)	Phosphatidylcholine	Lipids	1.09	3.50E-03	1.31E-02	1.45
43	PC(40:7)	Phosphatidylcholine	Lipids	1.78	2.56E-04	1.89E-03	1.43
44	L-Methionine	Methionine metabolism	Amino acid	1.46	4.61E-03	1.58E-02	1.41
45	Valyl-Lysine	Valine and lysine metabolism	Amino acid	1.96	4.09E-05	4.92E-04	1.39
46	PC(34:1)	Phosphatidylcholine	Lipids	1.19	1.58E-02	3.90E-02	1.39
**47**	**PC(33:3)**	**Phosphatidylcholine**	**Lipids**	**1.38**	**9.15E-03**	**2.62E-02**	**1.38**
48	Orotidine	Pyrimidine metabolism	Nucleosides and related	1.60	4.59E-03	1.58E-02	1.37
49	Glutamyl-valine	Valine metabolism	Amino acid	1.06	1.08E-02	2.96E-02	1.36
**50**	**3-Phospho-D-glycerate**	**Pentose phosphate**	**Carbohydrates and related**	**1.39**	**3.02E-03**	**1.18E-02**	**1.36**
**51**	**L-Acetylcarnitine**	**Fatty acid metabolism**	**Lipids**	**1.97**	**1.09E-03**	**5.50E-03**	**1.35**
52	Serine	Serine metabolism	Amino acid	1.88	1.42E-04	1.24E-03	1.34
53	L-glutamic acid	Arginine metabolism	Amino acid	1.16	1.93E-02	4.46E-02	1.34
**54**	**PA(34:1)**	**Phosphatidic acid**	**Lipids**	**1.92**	**2.06E-03**	**8.80E-03**	**1.33**
55	Histidinyl-Tryptophan	Tryptophan metabolism	Amino acid	1.41	1.01E-02	2.83E-02	1.33
**56**	**Cysteine-S-sulfate**	**Cysteine and methionine metabolism**	**Amino acid**	**1.84**	**8.77E-04**	**4.73E-03**	**1.33**
**57**	**Nicotinamide riboside**	**Nicotinate and nicotinamide metabolism**	**Energy metabolism**	**2.10**	**2.60E-05**	**3.57E-04**	**0.75**
58	PC(38:4)	Phosphatidylcholine	Lipids	2.34	1.34E-06	4.54E-05	0.75
59	L-Fucose	Fructose metabolism	Carbohydrates and related	1.47	1.08E-02	2.98E-02	0.74
60	PC(34:3)	Phosphatidylcholine	Lipids	1.51	1.76E-04	1.45E-03	0.74
61	PE(34:1)	Phosphatidylethanolamine	Lipids	2.47	2.82E-06	7.19E-05	0.74
**62**	**PC(36:2)**	**Phosphatidylcholine**	**Lipids**	**2.49**	**3.44E-07**	**1.77E-05**	**0.73**
63	Heptadecanoyl carnitine	Fatty acid metabolism	Lipids	1.01	5.73E-03	1.87E-02	0.72
64	Glycolic acid	Glyoxylate and dicarboxylate metabolism	Carbohydrates and related	1.14	4.54E-04	2.91E-03	0.72
65	PC(41:3)	Phosphatidylcholine	Lipids	2.21	8.78E-08	1.01E-05	0.72
66	PE(38:5)	Phosphatidylethanolamine	Lipids	1.92	8.44E-03	2.47E-02	0.71
**67**	**Trigonelline**	**Nicotinate and nicotinamide metabolism**	**Energy metabolism**	**2.17**	**1.71E-05**	**2.69E-04**	**0.71**
**68**	**L-Tryptophan**	**Tryptophan metabolism**	**Amino acid**	**2.50**	**2.47E-07**	**1.38E-05**	**0.71**
69	9-octadecenoic acid	Lineolic acid metabolism	Lipids	1.69	3.72E-04	2.49E-03	0.71
70	9-HODE	Lineolic acid metabolism	Lipids	1.58	9.31E-04	4.93E-03	0.70
**71**	**6-Hydroxynicotinate**	**Nicotinate and nicotinamide metabolism**	**Energy metabolism**	**1.95**	**3.20E-06**	**7.76E-05**	**0.67**
72	Decanoic acid	Fatty acid biosynthesis	Lipids	1.65	3.11E-04	2.17E-03	0.66
**73**	**Xanthine**	**Purine metabolism**	**Nucleosides and related**	**1.74**	**5.64E-06**	**1.14E-04**	**0.65**
**74**	**Nicotinate**	**Nicotinate and nicotinamide metabolism**	**Energy metabolism**	**2.51**	**3.91E-06**	**8.85E-05**	**0.65**
75	PC(42:10)	Phosphatidylcholine	Lipids	2.23	1.07E-06	3.93E-05	0.64
76	L-taurine	Taurine metabolism	Amino acid	1.34	7.87E-04	4.37E-03	0.64

**77**	**NAM**	**Nicotinate and nicotinamide metabolism**	**Energy metabolism**	**2.87**	**2.16E-06**	**6.04E-05**	**0.62**
78	Malonic acid	Fatty acid metabolism	Lipids	1.40	5.88E-05	6.41E-04	0.61
79	PC(42:9)	Phosphatidylcholine	Lipids	1.97	7.44E-03	2.27E-02	0.60
**80**	**3-Indoleacetonitrile**	**Tryptophan metabolism**	**Amino acid**	**1.86**	**6.57E-03**	**2.07E-02**	**0.59**
**81**	**SM C18:1**	**Phosphosphingolipids**	**Lipids**	**2.64**	**1.12E-06**	**4.05E-05**	**0.59**
82	Jasmonic acid	Linolenic acid metabolism	Lipids	1.84	2.52E-04	1.88E-03	0.57
**83**	**3-Hydroxy-L-kynurenine**	**Tryptophan metabolism**	**Amino acid**	**1.48**	**8.69E-03**	**2.52E-02**	**0.48**
**84**	**PC(38:6)**	**Phosphatidylcholine**	**Lipids**	**2.20**	**3.18E-08**	**6.81E-06**	**0.42**
**85**	**Quinolinate**	**Tryptophan metabolism**	**Amino acid**	**1.89**	**2.03E-04**	**1.61E-03**	**0.42**
**86**	**L-Kynurenine**	**Tryptophan metabolism**	**Amino acid**	**2.20**	**2.32E-07**	**1.35E-05**	**0.36**
**87**	**PE(38:6)**	**Phosphatidylethanolamine**	**Lipids**	**2.11**	**5.32E-04**	**3.27E-03**	**0.31**
88	Theophylline	Purine derivatives	Nucleosides and related	1.50	3.92E-03	1.41E-02	0.26
89	Theobromine	Purine derivatives	Nucleosides and related	1.10	6.16E-03	1.98E-02	0.26

Bold values indicate metaboites differentially expressed in both discovery and internal validation sets.

Table S6

The additional values of the RF-based 6-metabolite model beyond the FREEDOM clinical risk score in the external validation set.

	FREEDOM risk score + 6-metabolite panel				Reclassified
FREEDOM risk score	< 30%	30-60%	> 60%	Total	(%)
Patients not occurring MACEs, No.					
< 30% risk	187	27	1	215	13
30-60% risk	27	0	10	37	100
> 60% risk	1	0	1	2	50
Total	215	27	12		26
Patients occurring MACEs, No.					
< 30% risk	11	14	1	26	58
30-60% risk	0	4	15	19	79
> 60% risk	0	0	2	2	0
Total	11	18	18		64
Combined group					
< 30% risk	198	41	2	241	18
30-60% risk	27	4	25	56	93
> 60% risk	1	0	3	4	25
Total	226	45	30		32
	Estimate			*p* value	
Δ C-index	0.20 (0.12-0.28)			< 0.001	
NRI Categorical	0.60 (0.45-0.75)			< 0.001	
NRI Continuous	1.35 (1.14-1.55)			< 0.001	
IDI	0.27 (0.22-0.32)			< 0.001	

NRI: net reclassification improvement; IDI: integrated discrimination improvement.

References

1. Association AD. Diagnosis and classification of diabetes mellitus. *Diabetes Care* 2014; 37 Suppl 1:S81-90.
2. Otterstad JE. Measuring left ventricular volume and ejection fraction with the biplane Simpson's method. *Heart* 2002; 88(6):559-560.
3. Tuzcu EM, Berkalp B, De Franco AC, Ellis SG, Goormastic M, Whitlow PL et al. The dilemma of diagnosing coronary calcification: angiography versus intravascular ultrasound. *J Am Coll Cardiol* 1996; 27(4):832-838.
4. Sianos G, Morel MA, Kappetein AP, Morice MC, Colombo A, Dawkins K et al. The SYNTAX Score: an angiographic tool grading the complexity of coronary artery disease. *EuroIntervention* 2005; 1(2):219-227.
5. Gao R, Yang Y, Han Y, Huo Y, Chen J, Yu B et al. Bioresorbable Vascular Scaffolds Versus Metallic Stents in Patients With Coronary Artery Disease: ABSORB China Trial. *J Am Coll Cardiol* 2015; 66(21):2298-2309.
6. Grant R, Berg J, Mestayer R, Braidy N, Bennett J, Broom S et al. A Pilot Study Investigating Changes in the Human Plasma and Urine NAD+ Metabolome During a 6 Hour Intravenous Infusion of NAD. *Front Aging Neurosci* 2019; 11:257.
7. Rodenburg RJ. Biochemical diagnosis of mitochondrial disorders. *J Inherit Metab Dis* 2011; 34(2):283-292.
8. Bradford MM. A rapid and sensitive method for the quantitation of microgram quantities of protein utilizing the principle of protein-dye binding. *Anal Biochem* 1976; 72:248-254.
9. Bustin SA, Benes V, Garson JA, Hellemans J, Huggett J, Kubista M et al. The MIQE guidelines: minimum information for publication of quantitative real-time PCR experiments. *Clin Chem* 2009; 55(4):611-622.
10. Schmittgen TD, Livak KJ. Analyzing real-time PCR data by the comparative C(T) method. *Nat Protoc* 2008; 3(6):1101-1108.
11. Wang XB, Cui NH, Liu X, Liu X. Joint effects of mitochondrial DNA4977 deletion and serum folate deficiency on coronary artery disease in type 2 diabetes mellitus. *Clin Nutr* 2020; 39(12):3771-3778.
